# Secondary structure and domain architecture of the 23S and 5S rRNAs

**DOI:** 10.1093/nar/gkt513

**Published:** 2013-06-14

**Authors:** Anton S. Petrov, Chad R. Bernier, Eli Hershkovits, Yuzhen Xue, Chris C. Waterbury, Chiaolong Hsiao, Victor G. Stepanov, Eric A. Gaucher, Martha A. Grover, Stephen C. Harvey, Nicholas V. Hud, Roger M. Wartell, George E. Fox, Loren Dean Williams

**Affiliations:** ^1^School of Chemistry and Biochemistry, Georgia Institute of Technology, Atlanta, GA 30332, USA, ^2^Center for Ribosomal Origins and Evolution, Georgia Institute of Technology, Atlanta, GA 30332, USA, ^3^School of Chemical and Biomolecular Engineering, Georgia Institute of Technology, Atlanta, GA 30332, USA, ^4^Department of Biology and Biochemistry, University of Houston, Houston, TX 77204, USA and ^5^School of Biology, Georgia Institute of Technology, Atlanta, GA 30332, USA

## Abstract

We present a *de novo* re-determination of the secondary (2°) structure and domain architecture of the 23S and 5S rRNAs, using 3D structures, determined by X-ray diffraction, as input. In the traditional 2° structure, the center of the 23S rRNA is an extended single strand, which in 3D is seen to be compact and double helical. Accurately assigning nucleotides to helices compels a revision of the 23S rRNA 2° structure. Unlike the traditional 2° structure, the revised 2° structure of the 23S rRNA shows architectural similarity with the 16S rRNA. The revised 2° structure also reveals a clear relationship with the 3D structure and is generalizable to rRNAs of other species from all three domains of life. The 2° structure revision required us to reconsider the domain architecture. We partitioned the 23S rRNA into domains through analysis of molecular interactions, calculations of 2D folding propensities and compactness. The best domain model for the 23S rRNA contains seven domains, not six as previously ascribed. Domain 0 forms the core of the 23S rRNA, to which the other six domains are rooted. Editable 2° structures mapped with various data are provided (http://apollo.chemistry.gatech.edu/RibosomeGallery).

## INTRODUCTION

**Figure 1.**
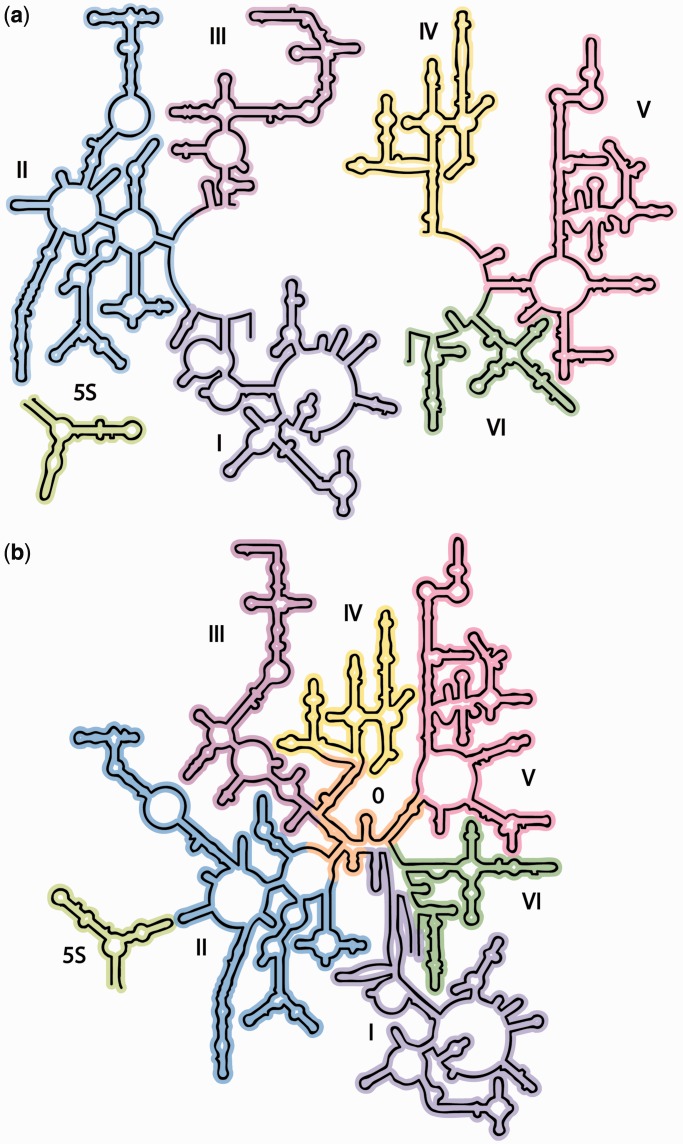
The 2° structures and domain architectures of the 23S and 5S rRNAs of *E. coli*. (**a**) The traditional 2° structure^phylo^, which is sheared into two fragments, and contains a central single-stranded region and six domains (Domain I, purple; Domain II, blue; Domain III, magenta; Domain IV, yellow; Domain V, pink; Domain VI, green), and (**b**) 2° structure^3D^, which accurately represents all helices, and contains seven domains (Domain 0 in orange; Domains I-VI are colored as in panel 1a). In 2° structure^phylo^, the central single-stranded region is partitioned between multiple domains, whereas in 2° structure^3D^, that same rRNA is double-helical and is fully contained within Domain 0. The 5S rRNA is light green and is placed in the proximity of Domain II in 2° structure^3D^ to reflect its position in three dimensions and its interactions with the 23S rRNA.

The ribosome, a macromolecular assembly of ribosomal RNAs (rRNAs) and ribosomal proteins (rProteins), synthesizes coded proteins in every cell of every organism. The ribosome comprises of large and small subunits that catalyze peptide bond formation (LSU) and decode mRNA (SSU). A key advance in understanding the ribosome was the determination of rRNA secondary structures (2° structures) by Brimacombe (1), Branlant (2) and Noller and Gutell (3). Noller and Gutell outlined the broadly appropriated ‘canonical’ 2° structure of the bacterial 23S rRNA (Figure 1a). Hundreds of 2° structures of rRNAs from a wide variety of organisms and organelles are available (3–10).

RNA 2° structures, with symbolic representations of base pairs, double-helices, loops, bulges and single-strands, provide frameworks for understanding structure, folding and function and for organizing a wide variety of information. RNA 2° structures reveal how local 2° elements are organized into quasi-independent domains, which can be used to infer mechanisms of global assembly and evolution. The depiction of the 23S rRNA 2° structure in Figure 1a illustrates how the LSU domain architecture has been traditionally defined (3–10).

A second key advance in understanding the ribosome was the determination of high-resolution 3D structures (10–18) from all three primary domains of the tree of life; Bacteria, Archaea and Eukaryota. The 3D structures of ribosomes confirm many aspects of the 2° structure of the 23S rRNA and validate the co-variation methods that were used for its construction.

However, critical differences distinguish the 2° structure of the 23S rRNA from 3D structures. The most significant discrepancy is in the heart of the 23S rRNA, which is represented by extended single-strands in the traditional 2° structure (Figure 1a). By contrast, 3D structures reveal this portion of the rRNA to be compact and double helical. Thus, the traditional 2° structure does not represent the correct helical configuration of the 23S rRNA. The incorrect representation of helices leads to downstream inaccuracies in the domain architecture.

Here, we present a *de novo* re-determination of the 2° structures of the 23S and 5S RNAs, using high resolution 3D structures as the input data. The goal is to establish 2° structures that are fully consistent with 3D structures and are as accurate and useful as possible. We seek to obtain 2° representations that allow facile conceptualization of vast available structural, functional and phylogenetic data. Previous revisions of the 23S rRNA 2° structure (19–22) that were proposed before determination of 3D structures do not reflect some important features of 23S rRNA. Our effort follows and extends discussions of improved 2° structures by Fox and Gutell (23) and by Leontis and Westhof (24,25).

We refer to our revision as 2° structure^3D^ (Figure 1b, the 2° structure derived from 3D data) to distinguish it from the traditional 2° structure^phylo^ (Figure 1a, the 2° structure derived primarily from phylogenetic data). The 2° structure^phylo^ contains six domains, each rooted in the extended single-stranded region at the center of the structure, and arbitrarily positions and orients the 5S rRNA. By contrast, 2° structure^3D^ contains seven domains; a central domain (Domain 0) forms the essential core of the 23S rRNA, to which the other six domains of the 23S rRNA are rooted. The 5S rRNA is positioned and oriented as an adjunct to Domain 2, based on its location and interactions in the 3D structure. The 2° structure^3D^ lacks the central single-stranded region of 2° structure^phylo^.

Representations of both LSU and SSU rRNAs are available online at http://apollo.chemistry.gatech.edu/RibosomeGallery so that others can evaluate and hopefully exploit and extend the revisions. We provide high-resolution editable versions of both 2° structure^3D^ and 2° structure^phylo^ mapped with a variety of data related to molecular interactions and geometry, phylogeny and evolution and partitioning of rRNA into helices and domains. These representations are available for *Escherichia coli*, *Thermus thermophilus*, *Haloarcula marismortui* and *Saccharomyces cerevisiae*. The work presented in this article is based on the bacterial 23S rRNA of *E. coli*, although the findings are applicable to the 23S and 28S rRNAs of other species.

## MATERIALS AND METHODS

### Data

The 2° structure^phylo^ (Figure 1a) was obtained from Noller at http://rna.ucsc.edu/rnacenter/ribosome_images.html. The 2° structure^3D^ was manually laid out, adjusted with the program XRNA (http://rna.ucsc.edu/rnacenter/xrna/xrna.html) and finalized with Adobe Illustrator. Atomic coordinates of the 23S rRNA of *E. coli* were obtained from the Nucleic Acid Databank (PDB ID: 3R8S) (11). Base-pairing and base-stacking interactions were obtained from the library of RNA interactions (FR3D) (26) and confirmed by inspection and in-house code. Mapping of data onto 2° structures was performed on the in-house RiboVision server http://apollo.chemistry.gatech.edu/RiboVision. Images of 3D structures were generated with PyMOL (27).

### Defining helices

To build the best 2° structural model of the 23S rRNA, we defined helices by specific geometric and interaction criteria. Helices contain paired bases. Optimum helical definitions maximize intra-helical base stacking and minimize inter-helical base stacking. For base pairing, we used the geometric criteria of Leontis and Westhof (28). We assumed that each nucleotide belongs uniquely to no more than one helix and that a helix contains contiguously stacked bases. The majority of helices obtained by this method agree with those obtained by Gutell (8). However, some of their helical boundaries required subtle revision. To minimize differences with 2° structure^phylo^, we left unaltered the incorrectly assigned portions of rRNA in 2° structure^3D^ if they did not impact the domain model. For example, new helix 49b remains represented by a loop in structure^3D^, as it is in structure^phylo^. We incorporated new Helices 25a, 26a, 49a and 49b.

### Phylogenetic conservation

Sequence conservation across all phylogeny was used to evaluate the generality of various helix and domain models. We aligned 23S rRNA/28S sequences from 122 organisms (29) intended to represent, as far as available data allow, a broad sampling of the phylogenetic tree of life including all three domains of life. Sequences came from 19 eukaryotic species, 67 bacterial species and 36 archaeal species. A complete list of organisms, strains and their taxonomic ID’s is given in Supplementary Table S1.

### Shannon entropy

A multiple sequence alignment of the 23S/28S rRNAs was used to calculate the fraction of nucleotide type *i* (C, G, A or U) in each position along the sequence. The probability (*p_i_*) of a nucleotide type at a given position was approximated by its fractional occupancy at that position in the aligned sequences. The Shannon Entropy (*H*) at each position was calculated from the probabilities *p_i_* according to Equation (1) (30,31).
(1)
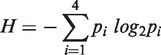



The Shannon Entropy ranges from 0 to 2. The minimum value indicates that the nucleotide type at that position is universally conserved. The maximum value corresponds to equivalent populations of all four nucleotide types.

### Defining domains

The 23S rRNA was partitioned into domains such that each helix is placed uniquely in one domain, using helices defined as described above. We used statistics on molecular interactions, and calculations of 2D folding propensities, compactness and sphericity to evaluate domain models. Inter- and intra-domain interactions include base-pairing, base-stacking and RNA–Mg^2+^–RNA interactions. First-shell Mg^2^^+^–RNA interactions are defined by Mg^2^^+^–RNA distance of <2.6 Å (32).

### Domain boundaries

The domain boundaries were defined by molecular interactions including base-pairing, base-stacking, base-phosphate and base-sugar interactions (listed here by priority). Domain boundaries were adjusted to minimize interactions between domains and to maximize interactions within domains. Each nucleotide belongs uniquely to one domain.

### Estimating the stability of the secondary structure of Domain 0

Mfold (33) was used to estimate stabilities of 2° structures. To convert Domain 0, for example, to a single RNA polymer *in silico*, the RNA fragments of Domain 0 were linked at the strand termini. Because Mfold cannot predict folds with non-canonical base pairs, Helix 26a was replaced with a canonical helix composed of Watson–Crick base pairs. The sequence of the substituted helix in the Mfold calculation is (5′GUAUAUGC3′:5′GCAUAUAC3′). The substitution is not expected to affect the energetics of the predicted folds, as the loop E motif and the substitute duplex have similar folding energies (34).

### Domain compactness and sphericity

We isolated putative domains from the 3D structure of the LSU and computed various characteristics of the isolated domains using the 3V software package (35). We estimated domain compactness with sphericity (36), which ranges from 0 to 1. Sphericity is calculated as SA_sphere_/SA_particle_ at fixed volume, where SA is surface area. A sphericity of 1 is most compact.

### Intra- and inter-domain interactions

We quantified molecular interactions of all of the rRNA, both helical and non-helical. RNA–RNA interaction frequencies within domains and between domains were estimated as a sum of base–base, phosphate–RNA and RNA–Mg^2^^+^–RNA interactions using network-based analysis (37–39). Local secondary interactions were excluded. Interaction matrices were constructed for 2° structure^phylo^ with six domains (a 6×6 matrix) and 2° structure^3D^ with seven domains (a 7×7 matrix). The diagonal elements of these matrices (*i,i*) describe the number of the intra-domain interactions for Domain *i*, whereas the off diagonal elements (*i,j*) contain the total number of interactions between Domain *i* and Domain *j*. To account for the difference in the numbers of nucleotides in various domains, the elements were scaled: the diagonal elements (*i,i*) were normalized to the number of nucleotides *N_i_* in Domain *i*, and the off-diagonal elements (*i,j*) were divided by an average number of nucleotides (*N_i_*+*N_j_*)/2 in Domains *i* and *j*.

## RESULTS

### 2° structure^phylo^

The 2° structure^phylo^ as proposed in 1981 (3) has been refined over time with increasingly sophisticated methods of co-variation analysis, combined with chemical probing data and information from 3D structures. The current 2° structure^phylo^ (Figure 1a) contains several bulges and helices that were absent in the 1981 2° structure (3) but inherits major features including the number of domains, the global layout of helices and domains, the central single-stranded region that roots each of the domains, and a separation into two halves. The central single-stranded region is fragmented between all six domains: Domain I (16–25 nt and 515–524 nt), Domain II (579–585 and 1255–1261), Domain III (1295–1298 and 1642–1645), Domain IV (1656–664 and 1997–2004), Domain V (2043–2054 and 2615–2625) and Domain VI (2630–2637 and 2781–2788).

### Defining Helix 26a

**Figure 2.**
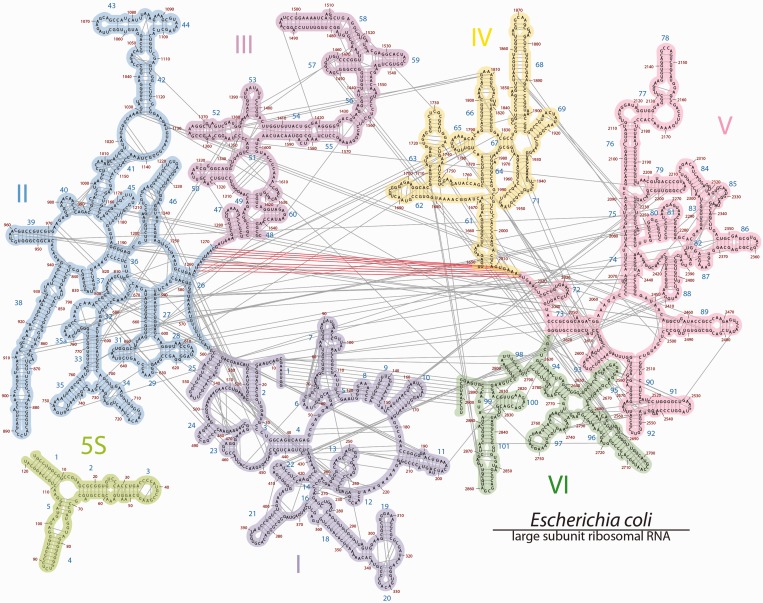
The 2° structure^phylo^ of the 23S and 5S rRNAs of *E. coli.* This ‘canonical’ 2° structure contains six domains colored as in Figure 1a. The central ‘singled-stranded’ region is partitioned to multiple domains. Helix numbers and nucleotide numbers are indicated. Nucleotides that are base paired in the 3D structure of the ribosome are connected by lines in the 2° structure here. The base-pair interactions within the central singled-stranded region (linking nucleotides A1262-C1270 to U2017-G2010) are highlighted in orange.

**Figure 3.**
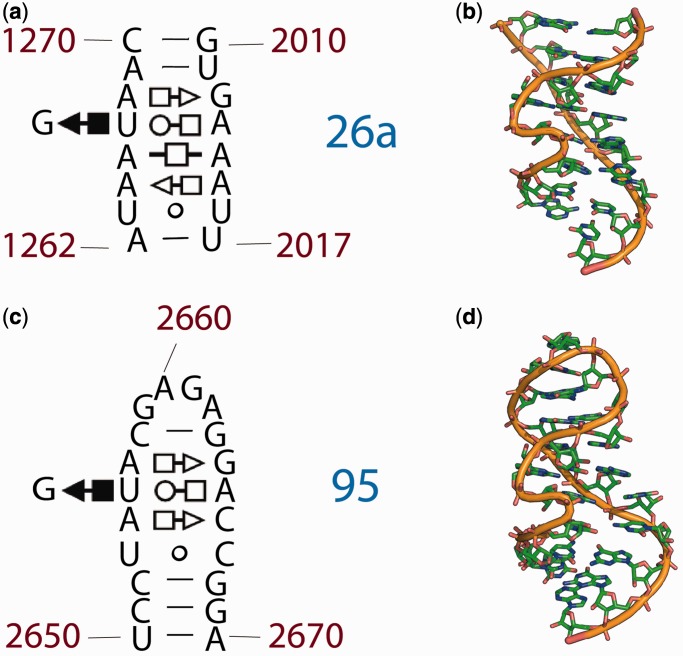
Loop E motifs: Helix 26a and Helix 95 of the 23S rRNA of *E. coli*. (**a**) The 2° structure and (**b**) the 3D structure of the rRNA that was traditionally represented as single-stranded, adapted from Leontis *et al.* (25,28). The symbols in the fragments of the 23S rRNA 2° structure represent non-Watson–Crick base pairs: circles correspond to the Watson–Crick edges, squares to the Hoogsteen edges, triangles to the sugar edges, the open symbols indicate *trans* basepairs and closed symbols, *cis* basepairs. (**c**) The 2° structure and (**d**) the 3D structure of the sarcin-ricin loop (Helix 95). A comparison the top and bottom panels illustrates the extent of 2° and 3D conservation of the loop E motif.

The projection of all base-pairing interactions onto 2° structure^phylo^ (Figure 2) illustrates that the central single-stranded region is involved in an intense network of molecular interactions and is not single stranded. The two halves of the extended single-stranded region are seen to associate by contiguous base-pairing interactions. Nucleotides 1262–1270 are paired with nucleotides 2010–2017, to form what we call Helix 26a. Inspection of the 3D structure confirms Helix 26a (Figure 3a and b), which was inferred previously by Fox and Gutell (23,40) and by Leontis and Westhof (24). Mutational studies support the importance of Helix 26a in the 23S rRNA (41). The center of Helix 26a contains non-canonical base pairs (28) U1263-U1216 (Watson–Crick/Watson–Crick), A1264-A1215 (trans Sugar edge/Hoogsteen), A1265-A1214 (trans Hoogsteen/Hoogsteen); U1267-A1213 (trans Watson–Crick/Hoogsteen); A1268-G2012 (trans Hoogsteen/Sugar edge) and U1267-G1266 (cis Hoogsteen/Sugar edge) with G1266 forming a triple base pair with U1267 and A1213 (Figure 3a). The central non-canonical region is flanked by canonical Watson–Crick base pairs of A1262 with U2017, A1269 with U2011 and C1270 with G2010 (24,25).

Inspection of Helix 26a in the 3D structure of the ribosome reveals that G1266 is extruded. This helix falls clearly within the parameters of the loop E motif (24,25). For comparison, an example of a different loop E motif is shown in Figure 3c and d.

We have aligned 23S/28S rRNA sequences from 122 organisms (29) that represent, as far as available data allow, a complete and non-biased sampling of the phylogenetic tree, including to all three domains of life. This alignment suggests that the pairing interactions of Helix 26a are conserved over the entire phylogenetic tree. In addition, we have inspected all available 3D structures of ribosomes (10–18) and observe that Helix 26a is conserved, as a helix, and more specifically, as a loop E motif, in each.

The alignment shows that the nucleotides of Helix 26a cluster into three groups according to the extent of conservation, as observed previously (25). The first conservation group is in the center of the helix and is characterized by conserved base identity and pairing mode: A1265-A1214 (trans Hoogsteen/Hoogsteen), U1267-A1213 (trans Watson–Crick/Hoogsteen), A1268-G2012 (trans Hoogsteen/Sugar edge) and U1267-G1266 (cis Hoogsteen/Sugar edge). The second conservation group contains 1264 nt paired with 2015 nt, with conserved trans Sugar edge/Hoogsteen interaction, but not conserved base identity. The third conservation group, at the termini of the helix, contains Watson–Crick pairs 1262–2017, 1269–2011 and 1270–2010. These base pairs conserve canonical pairing but not base identity. Sequence analysis of the sarcin ricin loop E motif (Helix 95, Figure 3c and d), also an loop E motif, reaffirms this pattern, with a single difference: the trans Hoogsteen–Hoogsteen A–A base pair in position 1264–2015 mutates in Bacteria to the less stable U–C base pair (25). In general, the sequence and pairing interactions of Helix 26a are more highly conserved than those of Helix 95. Conservation statistics for Helix 26a and Helix 95 are listed in the Supplementary Tables S2 and S3.

RNA melting experiments previously demonstrated that a loop E helix has stability comparable to that of a double-stranded Watson–Crick helix of a similar length (34). Loop E motifs have also been predicted and confirmed elsewhere in the 23S rRNA as well as in the 16S and 5S rRNA (24,25).

### Defining other helices

Using inspection of 3D structures and geometric calculations of interaction, we have partitioned the nucleotides of the 23S rRNA into helices. Each nucleotide and each base pair belongs uniquely to no more than one helix. Contiguously stacked bases are allocated to a common helix. A description of the method of partitioning of the nucleotides into helices is given in the ‘Materials and Methods’ section. The revised 2° structure^3D^ modestly expands the set of helices compared with 2° structure^phylo^. The 2° structure^3D^ contains Helices 25a, 26a, 49a and 49b that are absent from 2° structure^phylo^. The nucleotides within each of these helices are given in Table 1. Helix 25a was noted previously in *Haloarcula Marismortiu* (16) and *E. coli* (42). The definitions of helices in 2° structure^phylo^ are available from Gutell (8) at http://www.rna.icmb.utexas.edu/CAR/1A/.

**Table 1.** New helices in 2° structure^3D^

Helix	Nucleotides
Helix 25a	562–578
Helix 26a	1262–1270, 2010–2017
Helix 49a	1611–1620
Helix 49b	1309–1313, 1603–1605

### Re-defining 23S rRNA domains

#### Domain criteria

A domain is defined here as a compact and modular structure, stabilized by a self-contained nexus of molecular interactions that suggest the ability to fold autonomously. Each rRNA helix is considered to be autonomous and to belong uniquely to a single domain. We use computation and inspection to infer which regions of rRNA best satisfy these criteria. We use statistics of molecular interactions, 2D folding simulations and calculations of compactness and sphericity to evaluate domain models.

#### Domain criteria and 2° structure^phylo^

The 2° structure^phylo^ partitions several helices into multiple domains and therefore is not consistent with our criteria for a domain. The domain structure of 2° structure^phylo^ is not optimized to conform to observed networks of molecular interactions. Helix 26a in 2° structure^phylo^ is represented as single-stranded RNA and is partitioned between Domains II, IV and V. Helix 1, formed by the pairing of rRNA of the 3′ and the 5′ termini, is partitioned in 2° structure^phylo^ simultaneously into Domains I and VI.

#### Previous rRNA domain revisions

Fox and Gutell previously suggested that the 23S rRNA might contain a central core region forming a basic scaffold from which the domains emerge (23). Their central core, proposed before the determination of 3D structures of ribosomes, consists of Helices 26a, 26, 47, 48, 61, 72 and 73 (our numbering scheme). The Fox and Gutell proposal was, to our knowledge, never incorporated into a secondary representation or a domain model of the 23S rRNA.

#### Domain 0 and 2° structure^3D^

**Figure 4.**
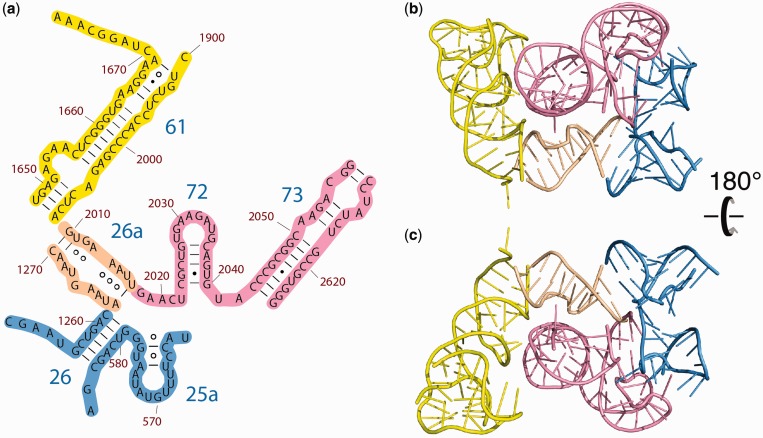
Domain 0, the central core of the 23S rRNA, to which all other 23S rRNA domains are rooted. (**a**) 2° Structure of Domain 0. The helices are numbered and distinguished by color. The coloring of helices is consistent with Figure 2. Helix 26a, with an extruded G and non-canonical base pairs, is orange. (**b**) The 3D structure of Domain 0, taken from the X-ray structure of the LSU, with the same helical coloring scheme as in (a). (**c**) Domain 0 rotated by 180° relative to (b).

**Figure 5.**
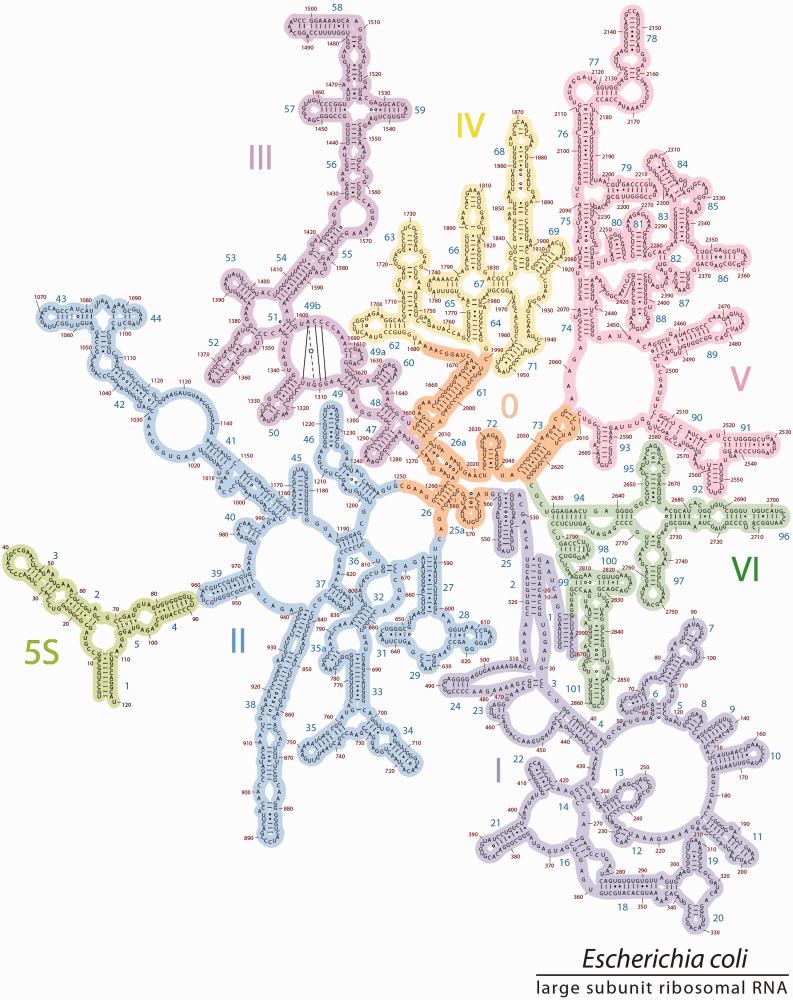
The 2° structure^3D^. The revised 2° structure of the 23S and 5S rRNAs of *E. coli*, is consistent with 3D structures. Domain 0 (orange) forms the central core of the 23S rRNA, to which all other domains are rooted. Domains 0–VI are colored as in Figure 1b. The 5S rRNA is placed in proximity to Helix 39 to reflect their locations in 3D space. The sequences of 23S and 5S rRNAs, the helix numbers and the domains are indicated. To preserve the traditional style of the 23S rRNA layout, Helix 49b is represented by base pairing lines across a loop in Domain III.

A subset of rRNA within the core of the LSU appears to be compact and autonomous and fits criteria for a domain. This subset of the 23S rRNA is called Domain 0. To conceptually create Domain 0 from the domains of the traditional model, we formed Helices 25a and 26a from single strands and appropriated Helix 26 from Domain II, Helix 61 from Domain IV and Helices 72 and 73 from Domain V (Figures 4 and 5, Table 2). The apparently single-stranded extension on Helix 61 (Figure 4a) is tightly coiled onto other elements of the Domain 0 (Figure 4b and c). Similarly, the apparently single-stranded extensions of Helix 26 fold back on each other and on the terminus of Helix 26. The location and interactions of Domain 0 with other domains are illustrated in 3D in the Supplementary Figures S1 and S2. Domain 0 corresponds well with the ancestral core of the LSU in the Bokov and Steinberg model of ribosomal evolution (42).

**Table 2.** Domain definitions of 2° structure^3D^

Domain	Nucleotides
Domain 0	562–586, 1251–1270, 1648–1678, 1990–2057, 2611–2625
Domain I	1–561, 2895–2904
Domain II	587–1250
Domain III	1271–1647
Domain IV	1679–1989
Domain V	2058–2610
Domain VI	2626–2894

The selection of Helices 25, 26, 26a, 61, 72 and 73 as elements of Domain 0 is based on several criteria. The domain elements are tightly networked with each other and are less integrated with surrounding rRNA. The six helices of Domain 0 are linked by 16 interlocking interactions (Supplementary Table S4). Helix 26a forms a total of one base pair, three base-phosphate or sugar-phosphate and one RNA–Mg^2^^+^–RNA interactions with Helices 26, 61 and 73. Helices 61 and 73 are further stabilized by interactions with each other. Helix 25a intercalates between Helices 72 and 73 and also forms an A-minor interaction with Helix 26 (42). Helix 72 interacts with Helix 73. By contrast, interactions of Helices 26, 61 and 73 with their (traditional) domains of origin are limited to terminal regions of the rRNA elements. Domain 0 appears to play a structural role. It contains a cleft that embraces the A- and P-regions of the peptidyl transfer center (PTC) and holds them in proximity.

Fox and Gutell’s Helices 47 and 48 are not included in Domain 0 because they are separate from it in three dimensions and are integrated by molecular interactions into Domain III. Thus, Helices 47 and 48 are allocated to Domain III in 2° structure^3D^ (Figure 5 and Supplementary Figure S3). Helix 25a, which was not identified in the Fox and Gutell model, is included in Domain 0. Helix 1 is wholly contained within Domain I.

The 5S rRNA is placed in proximity to Helix 39 to reflect their locations in 3D space and molecular interactions between the two rRNAs (Supplementary Figure S4). Modest adjustments to previous domains are implemented, but as far as possible, differences between 2° structure^3D^ and 2° structure^phylo^ are minimized.

#### Domain 0 is predicted to be an independent fold

Results from the program Mfold (33) suggest that Domain 0 is an autonomous folding unit. Mfold suggests that Domain 0 alone folds to the same secondary state as in the intact LSU. As Mfold can only predict folding of RNA containing canonical pairs, the sequence of Helix 26a was converted to a duplex of standard base pairs: 5′GUAUAUGC3′:5′GCAUAUAC3′. Helix 25a was not predicted correctly by Mfold because it contains alternating canonical and non-canonical base pairs. Folding of Domain 0 alone by Mfold, with the substituted Helix 26a gave the 2° structure observed within the intact LSU (See Supplementary Figure S5). In all, 43 of 49 base pairs in the secondary structure of Domain 0 (Supplementary Figure S5a) are correctly predicted by Mfold (Supplementary Figure S5b). The AU and GU base pairs at one end of Helices 72 and 73 are wrongly predicted. One of these base pairs involves an A that can interact with two possible U’s. Three closing base pairs of the hairpin-loop of Helix 61 are also wrongly predicted. However, this discrepancy results from a non-canonical base pair at this location. In general, the 2° structure of Domain 0 is well-predicted by Mfold, supporting the view that it is a distinct and structurally independent domain.

#### The 2°structure^3D^: sphericity and compactness

To evaluate domain models of the 23S rRNA, we estimated the compactness of each domain by computing its sphericity. A sphericity of one is exactly spherical and most compact (43). The average sphericity of Domains 0-VI of 2° structure^3D^ (0.235) is slightly greater than that of Domains I-VI of 2° structure^phylo^ (0.223). Sphericities of each domain are presented in Supplementary Table S5 (Domains I-VI of 2° structure^phylo^) and Supplementary Table S6 (Domains 0-VI of 2° structure^3D^). On average, the domains of 2° structure^3D^ are slightly more compact than those of 2° structure^phylo^.

#### The 2° structure^3D^: secondary and tertiary interactions

An optimized 2° structure would tend to give the greatest number of 2° interactions and the fewest tertiary interactions. An optimized domain model would tend to give the greatest number of intra-domain interactions and the fewest inter-domain interactions.

**Figure 6.**
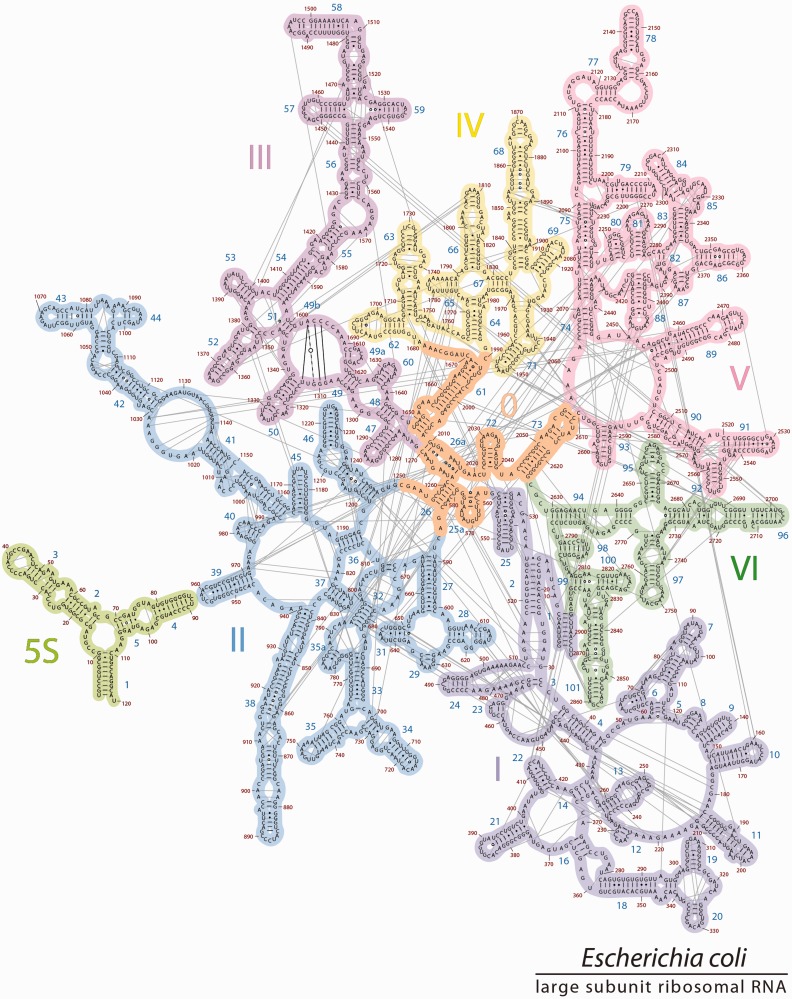
The mapping of all base pairing interactions onto 2° structure^3D^. Nucleotides that are base paired in the 3D structure of the ribosome are connected by lines in the 2° structure here. The 5S rRNA is placed in proximity to Helix 39 to reflect their relative locations in 3D space. The interactions between 23S and 5S rRNAa are illustrated in Supplementary Figure S4. Domain 0 is stabilized by many base-pairing interactions. The coloring scheme of the domains is the same as in Figure 5. The most frequent subtypes of base pair interactions [cWW, tWW, tSS and cSS, defined by Leontis (26)] are illustrated in Supplementary Figure S6.

The allocation of RNA–RNA interactions between secondary and tertiary interactions differs between 2° structure^3D^ and Structure^phylo^. Twenty-one tertiary interactions in 2° structure^phylo^ are converted to secondary interactions in 2° structure^3D^. This difference can be seen in the projections of all base-pairing interactions onto 2° structure^3D^ (Figure 6) and 2° structure^phylo^ (Figure 2). The short lines in Figures 2 and 6 that connect two opposing stands in a helix represent secondary interactions. Longer lines depict tertiary interactions. Eight base-pairing interactions of Helix 26a (residues 1262–1270 are paired with residues 2010–2017, with residue 1266 forming a triple base pair) are tertiary interactions in 2° structure^phylo^ and secondary interactions in 2° structure^3D^. Eight base-pairing interactions in Helix 1 (residues 1–8 are paired with 2895–2902) and 5 base-pairing interactions in Helix 2 (residues 26–30 are paired with 510–514) are represented as tertiary interactions in 2° structure^phylo^, but as secondary interactions in 2° structure^phylo^.

#### The 2° structure^3D^: inter and intra domain molecular interactions

We tabulated RNA–RNA interactions (estimated as the sum of the number of base–base, phosphate–RNA and RNA–Mg^2+^–RNA interactions) between and within domains for both 2° structure^3D^ and 2° structure^phylo^. Inter-domain interaction frequencies were scaled by the mean of the number of nucleotides in the two domains. The numerical values of both unscaled (Supplementary Tables S7 and S9) and scaled (Supplementary Tables S8 and S10) interactions for the 2° structure^phylo^ and 2° structure^3D^, as well as the detailed list of interactions between Domain 0 and Domains I–VI (Supplementary Table S11) are given in the Supplementary Data.

**Figure 7.**
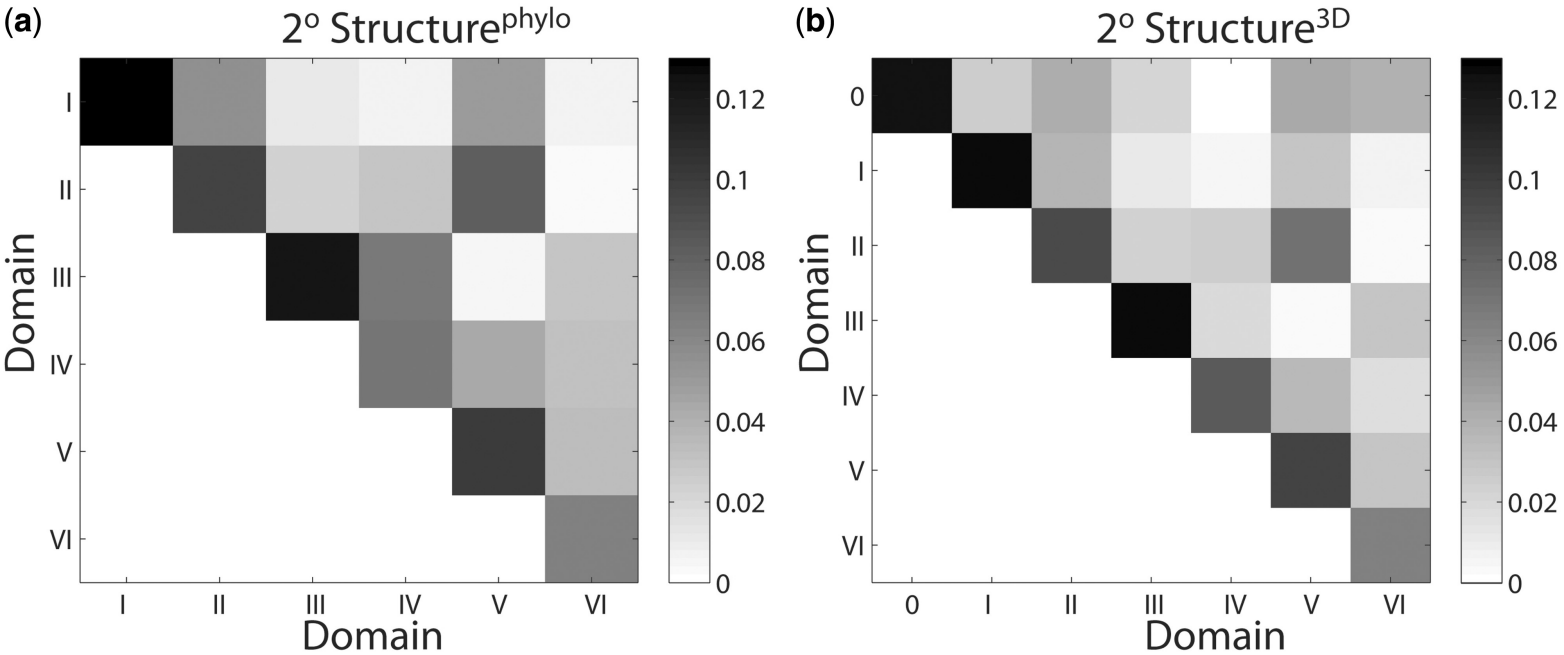
Molecular interactions within the LSU, partitioned by rRNA domain for (**a**) 2° Structure^phylo^ and (**b**) 2° Structure^3D^. The vertical and horizontal axes display the domain numbers. Scaled interaction frequencies were determined as the sum of the number of base–base, phosphate–RNA and RNA–Mg^2+^–RNA interactions, normalized by the average number of nucleotides in the domain pairs. Interaction frequencies were determined within domains (diagonal) and between domains (off-diagonal). The degree of shading indicates the frequencies of interaction within or between domains. The numerical values of the frequencies of interaction are given in Supplementary Tables S8 and S10.

The scaled interaction frequencies are presented for 2° structure^phylo^ (Figure 7a, Supplementary Table S8) and 2° structure^3D^ (Figure 7b, Supplementary Table S10). For both 2° structure^3D^ and 2° structure^phylo^, the number of scaled intra-domain interactions (diagonal elements) is always greater than the number of inter-domain interactions for a given domain (off-diagonal elements). Therefore, the interaction frequencies for the domains including Domain 0 are consistent with independent autonomous structural units. Comparison of the sum of diagonal elements in Structure^phylo^ (Supplementary Table S7) and 2° structure^3D^ (Supplementary Table S9) and 2° reveals that the total number of the unscaled intra-domain RNA–RNA interactions is slightly higher in 2° structure^3D^ (289) than 2° structure^phylo^ (287). This difference suggests the seven structural domains of 2° structure^3D^ are somewhat more independent than six 2° domains of 2° structure^phylo^, although the difference is small. It can be concluded that the 2° structure^3D^ is certainly not inferior to 2° structure^phylo^ by these criteria.

## DISCUSSION

### Revised 23S rRNA 2° structure

Our goal is to obtain 2° representations that allow facile conceptualization and organization of molecular interactions, 3D architecture, phylogeny and function. We have investigated the utility of 2° structure^3D^ and 2° structure^phylo^. The combined results demonstrate a greater accuracy and utility of 2° structure^3D^ over 2° structure^phylo^.

### Helices

We have performed an accurate and self-consistent partitioning of the 23S rRNA into helices. rRNA that is double stranded in the 3D structure is double stranded in 2° structure^3D^. A region of contiguous base pairs near the center of the rRNA is represented as Helix 26a rather than as the extended single strands in the traditional 2° structure^phylo^. Helix 26a appears to be critical to ribosomal structure and function, as it is conserved in all three major domains of the tree of life. The importance of Helix 26a is shown by conservation of base pairing in rRNA sequences that represent the most complete available sampling of the phylogenetic tree (Supplementary Table S1) (29), and by conservation of 3D structure.

### Domain models

Consistent treatment of helices as integral and indivisible elements of domains compels a revision of the global domain architecture. The revised domain model includes seven domains, rather than the traditional six. A central domain (Domain 0) forms the core of the 23S rRNA, to which the other six domains are rooted. The previous domain architecture (2° structure^phylo^) regards Helix 26a as two single strands that are carved between multiple domains, treating this helix anomalously from other helices. In the revised domain architecture (2° structure^3D^), Helix 26a, like other helices, is integrated into a single domain.

Using a consistent structure-based definition of helices, along with (i) calculations of 2° structure-folding propensities, (ii) calculations of sphericity and compactness and (iii) networking analysis of molecular interactions, we have formulated an improved domain model for the 23S rRNA, containing a central core domain (Domain 0). Folding algorithms suggest that Domain 0 is an autonomous folding unit. Domain 0 is compact, showing the greatest sphericity of any domain in either model (Supplementary Tables S5 and S6). Domain 0 is among the most highly networked domain of either model (Supplementary Tables S8 and S10, secondary interactions excluded), with a greater scaled frequency of intradomain molecular interactions.

### Assessing utility of domain models: projection of distance from the peptidyl transfer center

**Figure 8.**
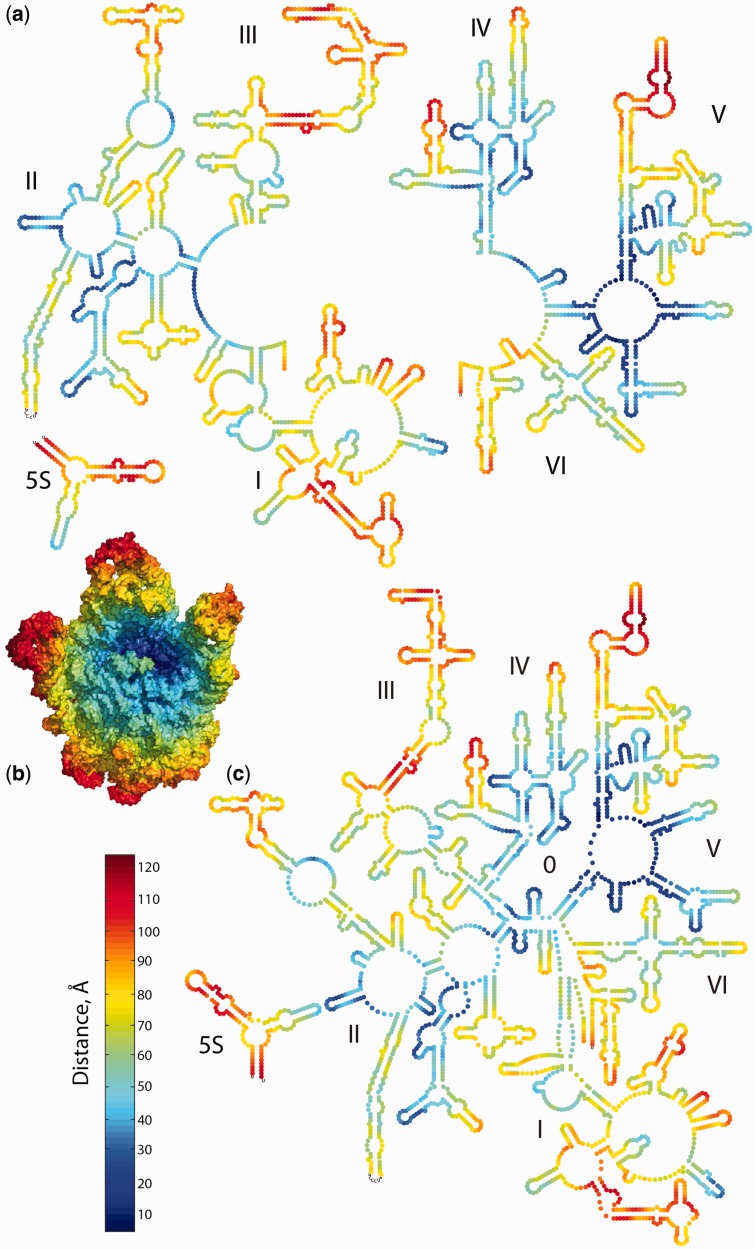
The 23S rRNA and 5S rRNA as a fine-grained onion. Nucleotides are colored by their distance, in the 3D structure of the LSU, from the site of peptidyl transfer. Nucleotides that are close to the site of peptidyl transfer are dark blue. Nucleotides that are remote from the PTC are red. (**a**) 2° Structure^phylo^ as an onion. Nucleotides are represented by circles. (**b**) The 3D structure of the LSU as an onion. The rRNA is in space-filling representation, with rProteins deleted for clarity. (**c**) 2° Structure^3D^ as an onion. The images in (a) and (c) are presented in independent images in Supplementary Figure S7.

Our goal is to advance a 2° structure and a domain model of the 23S rRNA that is coherent with the 3D structure. Projecting data from the 3D structure onto the 2° structures allows us to evaluate their coherence. When the distance (in Å) from the site of peptidyl transfer is projected onto 2° structure^phylo^ (Figure 8a), the relationship between 2° structure and 3D structure is tenuous and unconvincing. Nucleotides that are close together in 3D space, in this case closest to the PTC (dark blue in Figure 8b), are dispersed over 2° structure^phylo^. However, when these proximity data are projected onto 2° structure^3D^ (Figure 8c), the nucleotides closest together in 3D space are clustered in 2D space. The blue swatch running diagonally from lower left to upper right represents nucleotides that are near to the PTC (blue in Figure 8). Similarly, the nucleotides that are remote from the PTC (red in Figure 8) are incoherently distributed in 2° structure^phylo^ but are confined to the periphery of 2° structure^3D^. Therefore, the 2° structure^3D^ and the 3D structure present a consistent and comprehensible view of the 23S rRNA.

### Architectural similarities between the 23S and 16S rRNAs

As noted by Thirumalai, the domain structure of the LSU appears to differ significantly from that of the SSU (43). The LSU appears monolithic (16), whereas the SSU contains distinct and separate domains (44–46). However, 2° structure^3D^ suggests that the differences between the LSU and SSU are more modest than previously assumed. In 2° structure^3D^, the 23S rRNA, like the 16S rRNA, contains a central region to which other domains are rooted, i.e*.* both rRNAs have a central structural core. In 2° structure^3D^ of the 23S rRNA, like in the 2° structure of 16S rRNA, the structural center is distinct from the functional center (PTC in the 23S rRNA and the decoding center in 16S rRNA) but is in close proximity to it. These observations confirm and extend a previous hypothesis (23) that the apparent differences between the 23S and 16S rRNA are dependent on the domain model.

### What is a domain?

Ideally, an RNA domain is an independently stable globular structure composed of a continuous RNA strand, with domain boundaries defined by helicies. In the 2° structure of the 23S rRNA proposed in 1981 by Noller and Gutell (3), most 2° domains are continuous and are delimited by closing helices. However, those domain boundaries leave rRNA remnants, which were not assigned any domain. In more recent domain models (10), the remnants are included in proximal domains. The result is that none of the domains of 2° structure^phylo^ are delimited by a closing helix (Figure 1a). An additional difficulty with the concept of a RNA domain is one of scale: small RNA fragments such tetraloops (47,48) form independently stable and well-defined structures, closed by helices. Is a tetraloop a domain? A final difficulty with the concept of an RNA domain relates to possible continuity of RNA strands. A process by which older domains can be fragmented, and a mechanism of rRNA evolution, is suggested by rRNA expansion elements of eukaryotes. These expansion elements reveal that stem-loops, subdomains, and even domains can be inserted within previously established rRNA segments, fragmenting them. Considering that the ideal definition of a domain lacks universality, we did not impose helices as domain boundaries on 2° structure^3D^ and not restrain the RNA of a domain to be a continuous strand.

### Functional and evolutionary implications

**Figure 9.**
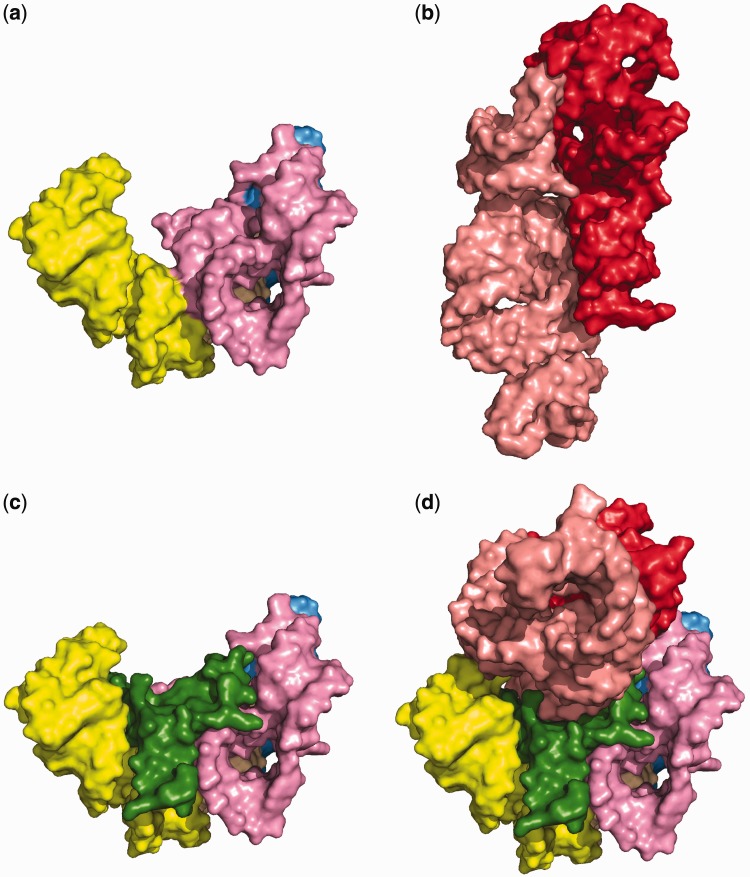
Space filling representations of (**a**) Domain 0. (**b**) The A-site and P-site rRNA of Domain V. (**c**) The association of Domain 0 with amino acids 113–168 of the ribosomal protein L3. (**d**) Domain 0 in association with rProtein L3 and the A-site and P-site region rRNA. Domain 0 helices are colored as in Figure 2a. rProtein L3 is green. The A-site rRNA is pink and P-site rRNA is red.

Domain 0 forms the entry and early portions of the exit tunnel, interacting with Domain V. Domain 0 forms a cradle (Figure 9a) for A-site and P-site of Domain V (Figure 9b). The interactions are mediated by a positively charged extension of rProtein L3 (amino acids 127–161) that is rich in arginines, lysines, and histidines and bears a net charge of +7 in *E. coli* (Figure 9c and d). The extension essentially fills a cavity between Domain 0 (Helices 61, 72, and 73) and the PTC (Helices 89 and 93) as shown in Figure 9d. The positively charged extension of rProtein L3 might be a fossil of one of the earliest non-coded products of the ancestral LSU.

Unlike the domains of 16S rRNA, which are predominantly isolated in space and ‘structurally autonomous’ (10), the domains of the 23S rRNA have convoluted shapes that interpenetrate in a monolithic assembly (16). These differences might be explained by a model in which early evolution of the LSU occurred in the absence of stabilizing peptides, which are thought to be a product of the primitive LSU (49). If so, stabilization of the early 23S rRNA was predominantly governed by cations (50,51) and RNA–RNA interactions (42), which resulted in interlocking motifs of the central core. By contrast the 16S may have originally assembled somewhat later, in the presence of short non-coded peptides made by the ancestral PTC.

## CONCLUSIONS

We have performed a *de novo* re-determination of the 2° structure of the 23S RNA using 3D structures as the primary data. We propose a revised 2° structure referred to as 2° structure^3D^. The 2° structure^3D^ lacks an extended central-stranded region in the heart of the 23S rRNA and contains seven 2° domains in contrast to six in the traditional 2° structure^phylo^. The 2° structure^3D^ contains a central core domain, which we call Domain 0, from which all other domains branch. Domain 0 is highly conserved over phylogeny and provides a structural integrity of 23S rRNA.

The 2° structure^3D^ of 23S rRNA of *E. coli*, as presented here, is readily generalized to other species. Along with the maps discussed in the manuscript, we have also generated 2° structures^3D^ for *T. thermophilus*, *H. marismortui*, and *S. cerevisiae.* Additionally, we have mapped the variety of data onto the 2° structure^3D^ and the conventional 2° structure^phylo^. On-line editable representations are available in a gallery at http://apollo.chemistry.gatech.edu/RibosomeGallery.

## SUPPLEMENTARY DATA

Supplementary Data are available at NAR Online: Supplementary Tables 1–11 and Supplementary Figures 1–7.

Supplementary Data
