# Supplementary material for: Secondary structure and domain architecture of the 23S and 5S rRNAs
Source: Nucleic Acids Res. 2013 Jun 14;41(15):7522–35. doi: 10.1093/nar/gkt513 (PMC3753638; doi:10.1093/nar/gkt513)
Supplement: Supplementary Data [file supp_41_15_7522__index.html]

Secondary structure and domain architecture of the 23S and 5S rRNAs — Secondary structure and domain architecture of the 23S and 5S rRNAs — Supplementary Data 

# Secondary structure and domain architecture of the 23S and 5S rRNAs

## 

files

**Files in this Data Supplement:**

- Supplementary Data - docx file
