# Supplementary material for: Secondary structure and domain architecture of the 23S and 5S rRNAs
Source: Nucleic Acids Res. 2013 Jun 14;41(15):7522–35. doi: 10.1093/nar/gkt513 (PMC3753638; doi:10.1093/nar/gkt513)
Supplement: Supplementary Data [file supp_gkt513_nar-00993-r-2013-File011.docx]

Secondary Structure and Domain Architecture of the 23S and 5S rRNAs

(Supplementary Data)

Anton S. Petrov^1^, Chad R. Bernier^1^, Eli Hershkovits^1^, Yuzhen Xue^2,^, Chris C. Waterbury^1^, Chiaolong Hsiao^1^, Victor G. Stepanov^3^, Eric A. Gaucher^4^, Martha A. Grover^2^, Stephen C. Harvey^1,4^, Nicholas V. Hud^1^, Roger M. Wartell^4^, George E. Fox^3^,and Loren Dean Williams^1*^

Center for Ribosomal Origins and Evolution, Georgia Institute of Technology, Atlanta, GA 30332, (1) School of Chemistry and Biochemistry, Georgia Institute of Technology, (2) School of Chemical and Biomolecular Engineering, Georgia Institute of Technology, (3) Department of Biology and Biochemistry, University of Houston, Houston, TX, 77204, (4) School of Biology, Georgia Institute of Technology.

*Corresponding author: loren.williams@chemistry.gatech.edu

**Table S1.** Sampling each of the three major branches of the tree of life: species names, strains, and NCBI tax id of 122 organisms^a^

| **Species** | **Strain** | **Tax ID** |
| --- | --- | --- |
| **Archaea** | | |
| Aeropyrum pernix | K1 | 272557 |
| Archaeoglobus fulgidus | DSM 4304 | 224325 |
| Caldivirga maquilingensis | IC-167 | 397948 |
| Haloarcula marismortui | ATCC 43049 | 272569 |
| Halobacterium sp. | NRC-1 | 64091 |
| Haloferax volcanii | DS2 | 309800 |
| Haloquadratum walsbyi | DSM 16790 | 362976 |
| Halorubrum lacusprofundi | ATCC 49239 | 416348 |
| Hyperthermus butylicus | DSM 5456 | 415426 |
| Ignicoccus hospitalis | KIN4/I | 453591 |
| Metallosphaera sedula | DSM 5348 | 399549 |
| Methanocaldococcus jannaschii | DSM 2661 | 243232 |
| Methanococcoides burtonii | DSM 6242 | 259564 |
| Methanococcus aeolicus | Nankai-3 | 419665 |
| Methanocorpusculum labreanum | Z | 410358 |
| Methanoculleus marisnigri | JR1 | 368407 |
| Methanopyrus kandleri | AV19 | 190192 |
| Methanoregula boonei | 6A8 | 456442 |
| Methanosaeta thermophila | PT | 349307 |
| Methanosarcina acetivorans | C2A | 188937 |
| Methanosarcina barkeri | Fusaro | 269797 |
| Methanosarcina mazei | Go1 | 192952 |
| Methanosphaera stadtmanae | DSM 3091 | 339860 |
| Methanospirillum hungatei | JF-1 | 323259 |
| Methanothermobacter thermautotrophicus | Delta H | 187420 |
| Nanoarchaeum equitans | Kin4-M | 228908 |
| Natronomonas pharaonis | DSM 2160 | 348780 |
| Picrophilus torridus | DSM 9790 | 263820 |
| Pyrobaculum calidifontis | JCM 11548 | 410359 |
| Pyrococcus furiosus | DSM 3638 | 186497 |
| Staphylothermus marinus | F1 | 399550 |
| Sulfolobus acidocaldarius | DSM 639 | 330779 |
| Sulfolobus tokodaii | 7 | 273063 |
| Thermococcus kodakarensis | KOD1 | 69014 |
| Thermofilum pendens | Hrk 5 | 368408 |
| Thermoplasma volcanium | GSS1 | 273116 |
| **Bacteria** | | |
| Acinetobacter sp. | RUH2624 | 575564 |
| Agrobacterium tumefaciens | C58 | 176299 |
| Anabaena variabilis | ATCC 29413 | 240292 |
| Azoarcus sp. | BH72 | 62928 |
| Bacillus anthracis | Ames | 198094 |
| Bacteroides thetaiotaomicron | VPI-5482 | 226186 |
| Bartonella henselae | Houston-1 | 283166 |
| Bifidobacterium longum | DJO10A | 205913 |
| Blochmannia floridanus |  | 203907 |
| Bradyrhizobium japonicum | USDA 110 | 224911 |
| Buchnera aphidicola | 5A | 563178 |
| Burkholderia sp. | 383 | 269483 |
| Caulobacter crescentus | NA1000 | 565050 |
| Chlamydophila caviae | GPIC | 227941 |
| Chlorobium tepidum | TLS | 194439 |
| Coxiella burnetii | RSA 493 | 227377 |
| Crocosphaera watsonii | WH 8501 | 165597 |
| Cytophaga hutchinsonii | ATCC 33406 | 269798 |
| Dechloromonas aromatica | RCB | 159087 |
| Dehalococcoides ethenogenes | 195 | 243164 |
| Deinococcus radiodurans | R1 | 243230 |
| Escherichia coli | DH1 | 536056 |
| Fusobacterium nucleatum subsp. nucleatum | ATCC 25586 | 190304 |
| Geobacillus kaustophilus | HTA426 | 235909 |
| Geobacter sulfurreducens | KN400 | 663917 |
| Gloeobacter violaceus | PCC 7421 | 251221 |
| Gluconobacter oxydans | 621H | 290633 |
| Haemophilus influenzae | Rd KW20 | 71421 |
| Helicobacter hepaticus | ATCC 51449 | 235279 |
| Legionella pneumophila | Lens | 297245 |
| Leifsonia xyli subsp. xyli | CTCB07 | 281090 |
| Listeria monocytogenes | EGD-e | 169963 |
| Magnetococcus sp. | MC-1 | 156889 |
| Magnetospirillum magnetotacticum | MS-1 | 272627 |
| Mesoplasma florum | L1 | 265311 |
| Mycobacterium leprae | TN | 272631 |
| Neisseria gonorrhoeae | FA 1090 | 242231 |
| Nitrosomonas europaea | ATCC 19718 | 228410 |
| Novosphingobium aromaticivorans | DSM 12444 | 279238 |
| Oceanobacillus iheyensis | HTE831 | 221109 |
| Photorhabdus luminescens subsp. laumondii | TTO1 | 243265 |
| Polaromonas sp. | JS666 | 296591 |
| Porphyromonas gingivalis | W83 | 242619 |
| Propionibacterium acnes | KPA171202 | 267747 |
| Pseudomonas aeruginosa | UCBPP-PA14 | 208963 |
| Ralstonia eutropha | JMP134 | 264198 |
| Rhodobacter sphaeroides | ATCC 17025 | 349102 |
| Rhodopirellula baltica | SH 1 | 243090 |
| Shewanella oneidensis | MR-1 | 211586 |
| Sinorhizobium meliloti | 1021 | 266834 |
| Staphylococcus aureus subsp. aureus | MRSA252 | 282458 |
| Streptococcus pneumoniae | R6 | 171101 |
| Streptomyces coelicolor | A3(2) | 100226 |
| Symbiobacterium thermophilum | IAM 14863 | 292459 |
| Synechococcus sp. | PCC 7335 | 91464 |
| Synechocystis sp. | PCC 6803 | 1148 |
| Thermoanaerobacter tengcongensis | MB4 | 273068 |
| Thermosynechococcus elongatus | BP-1 | 197221 |
| Thermotoga maritima | MSB8 | 243274 |
| Thermus thermophilus | HB8 | 300852 |
| Treponema pallidum subsp. pallidum | Nichols | 243276 |
| Tropheryma whipplei | Twist | 203267 |
| Vibrio cholerae O1 El Tor | N16961 | 243277 |
| Wolbachia endosymbiont | strain TRS of Brugia malayi | 292805 |
| Xanthomonas axonopodis pv. citri | 306 | 190486 |
| Yersinia pestis | CO92 | 214092 |
| Zymomonas mobilis subsp. mobilis | ATCC 10988 | 555217 |
| **Eukaryotes** | | |
| Anopheles gambiae | PEST | 180454 |
| Arabidopsis thaliana |  | 3702 |
| Caenorhabditis elegans |  | 6239 |
| Danio rerio |  | 7955 |
| Dictyostelium discoideum | AX4 | 352472 |
| Drosophila melanogaster |  | 7227 |
| Gallus gallus |  | 9031 |
| Giardia intestinalis | ATCC 50581 | 598745 |
| Guillardia theta |  | 55529 |
| Homo sapiens |  | 9606 |
| Mus musculus |  | 10090 |
| Oryza sativa japonica group |  | 39947 |
| Plasmodium falciparum | 3D7 | 36329 |
| Saccharomyces cerevisiae | S288c | 559292 |
| Schizosaccharomyces pombe | 972h- | 284812 |
| Tetrahymena thermophila | SB210 | 312017 |
| Thalassiosira pseudonana | CCMP1335 | 296543 |
| Trypanosoma brucei subsp. brucei | 927/4 GUTat10.1 | 999953 |
| Yarrowia lipolytica | CLIB122 | 284591 |

1. The sequences are from nineteen eukaryotic species, sixty-seven bacterial species, and thirty-six archaeal species.

**Table S2.** Conservation properties of Helix 26a based on the sequence alignment of 122 species^a^

| Nucleotide Number | Nucleotide Type^b^ | Consensus Nucleotide Type^c^ | Fraction of the Most Conserved Nucleotide | Shannon Entropy |
| --- | --- | --- | --- | --- |
| 1262 | A | A | 0.62 | 1.38 |
| 1263 | U | U | 0.58 | 1.28 |
| 1264 | A | G | 0.48 | 1.70 |
| 1265 | A | A | 0.99 | 0.07 |
| 1266 | G | G | 1.00 | 0.00 |
| 1267 | U | U | 1.00 | 0.00 |
| 1268 | A | A | 1.00 | 0.00 |
| 1269 | A | G | 0.70 | 0.87 |
| 1270 | C | C | 0.93 | 0.40 |
| 2010 | G | G | 0.93 | 0.40 |
| 2011 | U | U | 0.55 | 1.05 |
| 2012 | G | G | 0.98 | 0.08 |
| 2013 | A | A | 0.99 | 0.01 |
| 2014 | A | A | 0.97 | 0.20 |
| 2015 | A | A | 0.62 | 1.37 |
| 2016 | U | U | 0.60 | 1.28 |
| 2017 | U | U | 0.61 | 1.41 |

1. as listed in Table S1.
2. in *E.Coli.*
3. based on the alignment of 122 species listed in Table S1.

**Table S3.** Conservation of Helix 95 based on the sequence alignment of 122 species^a^

| Nucleotide Number | Nucleotide Type^b^ | Consensus Nucleotide Type^c^ | Fraction of the Most Conserved Nucleotide | Shannon Entropy |
| --- | --- | --- | --- | --- |
| 2650 | U | C | 0.46 | 1.78 |
| 2651 | C | C | 0.73 | 1.14 |
| 2652 | C | C | 0.53 | 1.42 |
| 2653 | U | U | 0.89 | 0.53 |
| 2654 | A | A | 0.98 | 0.14 |
| 2655 | G | G | 0.99 | 0.07 |
| 2656 | U | U | 0.99 | 0.07 |
| 2657 | A | A | 0.99 | 0.07 |
| 2658 | C | C | 0.99 | 0.07 |
| 2659 | G | G | 1.00 | 0.00 |
| 2660 | A | A | 1.00 | 0.00 |
| 2661 | G | G | 0.98 | 0.12 |
| 2662 | A | A | 0.98 | 0.08 |
| 2663 | G | G | 0.99 | 0.07 |
| 2664 | G | G | 1.00 | 0.00 |
| 2665 | A | A | 0.99 | 0.07 |
| 2666 | C | C | 0.55 | 1.13 |
| 2667 | C | C | 0.98 | 0.17 |
| 2668 | G | G | 0.64 | 1.51 |
| 2669 | G | G | 0.76 | 1.03 |
| 2670 | A | G | 0.47 | 1.74 |

1. as listed in Table S1.
2. in *E.Coli.*
3. based on the alignment of 122 species listed in Table S1.

**Table S4.** Tertiary intra-Domain 0 molecular interactions^a^

| **Nucleotide A** | **Helix M** |  | **Nucleotide B** | **Helix N** |
| --- | --- | --- | --- | --- |
| *Base-Base* | | | | |
| U562 | H25a |  | A2033 | H72 |
| A572 | H25a |  | G2029 | H72 |
| A2013 | H26a |  | U2613 | H73 |
| *Phosphate-RNA* | | | | |
| A575 | H25a |  | C2055 | H73 |
| G577 | H25a |  | A1254 | H26 |
| G578 | H25a |  | U1255 | H26 |
| G578 | H25a |  | U580 | H26 |
| U1648 | H61 |  | C1270 | H26a |
| A2019 | H26a |  | G579 | H26 |
| A2019 | H26a |  | U2017 | H26a |
| A2031 | H72 |  | U573 | H25a |
| G2049 | H73 |  | A1654 | H61 |
| G2049 | H73 |  | A2005 | H61 |
| G2618 | H73 |  | C2023 | H72 |
| *RNA-Mg^2+^-RNA* | | | | |
| A1268 | H26a |  | C2006 | H61 |
| A1265 | H26a |  | U2615 | H73 |

a) Pairwise base-base, phosphate-RNA, and RNA-Mg^2+^-RNA interactions between nucleotide A of helix M and nucleotide B of helix N.

**Table S5.** Areas, volumes, and sphericities of the six domains of 2° Structure^phylo^

| Domain | V(Å^3^) | A(Å^2^) | ψ |
| --- | --- | --- | --- |
| 1 | 180930 | 74516 | 0.21 |
| 2 | 205768 | 87801 | 0.19 |
| 3 | 117663 | 48543 | 0.24 |
| 4 | 107759 | 45531 | 0.24 |
| 5 | 181710 | 77721 | 0.2 |
| 6 | 85053 | 36581 | 0.26 |

**Table S6**. Areas, volumes, and sphericities of the seven domains of 2° Structure^3D^

| Domain | V(Å^3^) | A(Å^2^) | ψ |
| --- | --- | --- | --- |
| 0 | 50536 | 22240 | 0.3 |
| 1 | 180930 | 74516 | 0.21 |
| 2 | 192316 | 82633 | 0.19 |
| 3 | 117363 | 48543 | 0.24 |
| 4 | 90572 | 38214 | 0.26 |
| 5 | 161972 | 68874 | 0.19 |
| 6 | 85059 | 36581 | 0.26 |

**Table S7.** Frequencies of intra- and inter-domain molecular interactions^a^ of the 23S rRNA partitioned into six domains of 2° Structure^phylo^

|  | I | II | III | IV | V | VI |
| --- | --- | --- | --- | --- | --- | --- |
| I | 75 | 35 | 5 | 3 | 29 | 3 |
| II | 0 | 64 | 12 | 15 | 52 | 1 |
| III | 0 | 0 | 44 | 25 | 2 | 9 |
| IV | 0 | 0 | 0 | 26 | 21 | 10 |
| V | 0 | 0 | 0 | 0 | 61 | 15 |
| VI | 0 | 0 | 0 | 0 | 0 | 17 |

a) Pairwise base-base, phosphate-RNA, and RNA-Mg^2+^-RNA interactions between Domains *i* and *j*.

**Table S8.** Scaled frequencies of intra- and inter-domain molecular interactions^a^ of the 23S rRNA partitioned into six domains of 2° Structure^phylo^

|  | I | II | III | IV | V | VI |
| --- | --- | --- | --- | --- | --- | --- |
| I | 0.131 | 0.057 | 0.011 | 0.006 | 0.049 | 0.007 |
| II | 0 | 0.096 | 0.023 | 0.029 | 0.082 | 0.002 |
| III | 0 | 0 | 0.123 | 0.070 | 0.004 | 0.029 |
| IV | 0 | 0 | 0 | 0.071 | 0.043 | 0.031 |
| V | 0 | 0 | 0 | 0 | 0.101 | 0.034 |
| VI | 0 | 0 | 0 | 0 | 0 | 0.063 |

a) Pairwise base-base, phosphate-RNA, and RNA-Mg^2+^-RNA interactions between Domains *i* and *j*.

**Table S9**. Frequencies of intra- and inter-domain molecular interactions^a^ of the 23S rRNA partitioned into seven domains of 2° Structure^3D^

|  | 0 | I | II | III | IV | V | VI |
| --- | --- | --- | --- | --- | --- | --- | --- |
| 0 | 16 | 9 | 16 | 5 | 0 | 15 | 8 |
| I | 0 | 73 | 23 | 5 | 2 | 17 | 3 |
| II | 0 | 0 | 60 | 12 | 12 | 43 | 1 |
| III | 0 | 0 | 0 | 44 | 6 | 1 | 9 |
| IV | 0 | 0 | 0 | 0 | 26 | 15 | 5 |
| V | 0 | 0 | 0 | 0 | 0 | 53 | 12 |
| VI | 0 | 0 | 0 | 0 | 0 | 0 | 17 |

a) Pairwise base-base, phosphate-RNA, and RNA-Mg^2+^-RNA interactions between Domains *i* and *j*.

**Table 10S.** Scaled frequencies of intra- and inter-domain molecular interactions^a^ of the 23S rRNA partitioned into six domains of 2° Structure^3D^

|  | 0 | I | II | III | IV | V | VI |
| --- | --- | --- | --- | --- | --- | --- | --- |
| 0 | 0.125 | 0.026 | 0.041 | 0.021 | 0 | 0.044 | 0.040 |
| I | 0 | 0.128 | 0.038 | 0.011 | 0.005 | 0.030 | 0.007 |
| II | 0 | 0 | 0.093 | 0.024 | 0.025 | 0.072 | 0.002 |
| III | 0 | 0 | 0 | 0.126 | 0.018 | 0.002 | 0.029 |
| IV | 0 | 0 | 0 | 0 | 0.085 | 0.035 | 0.017 |
| V | 0 | 0 | 0 | 0 | 0 | 0.096 | 0.029 |
| VI | 0 | 0 | 0 | 0 | 0 | 0 | 0.063 |

a) Pairwise base-base, phosphate-RNA, and RNA-Mg^2+^-RNA interactions between Domains *i* and *j*.

**Table S11.** Inter-domain molecular interactions^a^ between Domain 0 and other domains

| DOMAIN 0-DOMAIN I | | | | |
| --- | --- | --- | --- | --- |
| *Base-Base* | | | | |
| U580 - A515 | C581 - A514 | U1258 - U448 | A1260 - A515 | U2041 - A529 |
| G2625 - A526 | G2018 - C531 | C2044 - A14 |  |  |
| *Phosphate-RNA* | | | | |
| C2043 - C527 |  |  |  |  |
| **DOMAIN 0-DOMAIN II** | | | | |
| *Base-Base* | | | | |
| U569 - A983 | A586 - G809 | A1254 - G808 | A2014 - U747 | G2027 - A981 |
| C2036 - A981 | A2037 - A980 |  |  |  |
| *Phosphate-RNA* | | | | |
| U566 - G809 | U568 - A945 | U569 - A945 | G570 - A972 | G1659 - A743 |
| A1676 - U741 | C2025 - U1130 | U2026 - U1130 | U2039 - G1136 |  |
| **DOMAIN 0-DOMAIN III** | | | | |
| *Phosphate-RNA* | | | | |
| U1647 - U1325 |  |  |  |  |
| *Mg-mediated* | | | | |
| U1647 - G1271 | U1647 - A1272 | U1648 - G1271 | U1648 - A1272 |  |
| **DOMAIN 0-DOMAIN IV** | | | | |
| None | | | | |
| **DOMAIN 0-DOMAIN V** | | | | |
| *Base-Base* | | | | |
| A2031 - G2455 | A2054 - A2577 |  |  |  |
| *Phosphate-RNA* | | | | |
| G570 - C2499 | A575 - C2499 | G577 - G2502 | U1255 - G2502 | U1255 - A2060 |
| A1669 - G2549 | G2032 - G2454 | G2032 - A2572 | A2614 - G2578 | C2611 - C2610 |
| *RNA-Mg^2+^-RNA* | | | | |
| U576 - A2503 | C1670 - G2550 | C2611 - C2579 |  |  |
| **DOMAIN 0-DOMAIN VI** | | | | |
| *Base-Base* | | | | |
| G1660 - U2689 | C2000 - U2689 | C2047 - A2823 |  |  |
| *Phosphate-RNA* | | | | |
| G1653 - G2822 | U1662 - U2687 | C1999 - C2723 | C2043 - G2777 | G2623 - A2826 |

*

a) Pairwise base-base, phosphate-RNA, and RNA-Mg^2+^-RNA interactions between nucleotide A of Domain 0 and nucleotide B of Domain N.


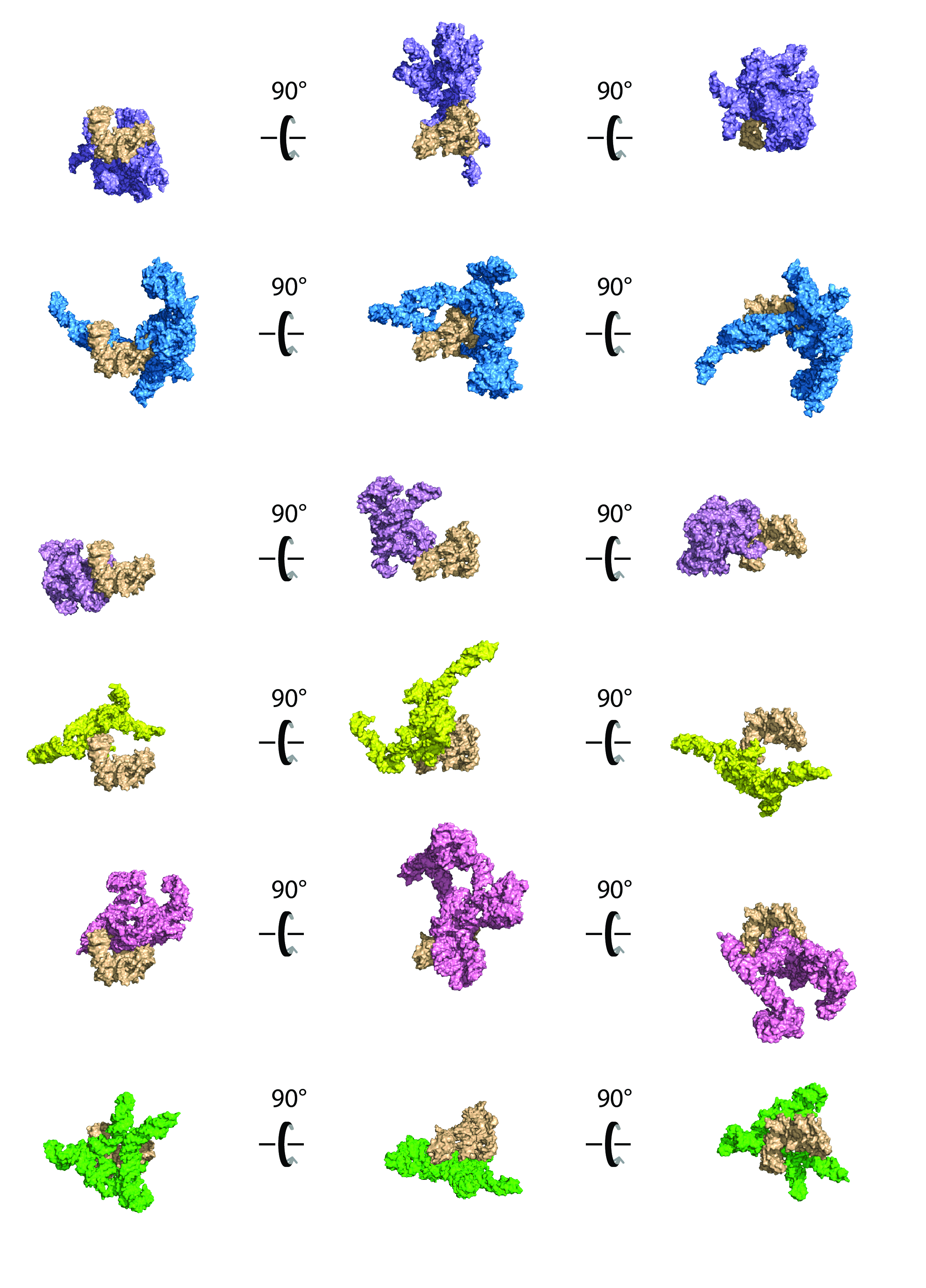


**Figure S1.** Domain 0 of 23S rRNA in association with individual Domains I-VI, rotated around the x-axis. Domain 0, tan; Domain I, purple; Domain II, blue; Domain III, magenta; Domain IV, yellow; Domain V, pink; Domain VI, green.


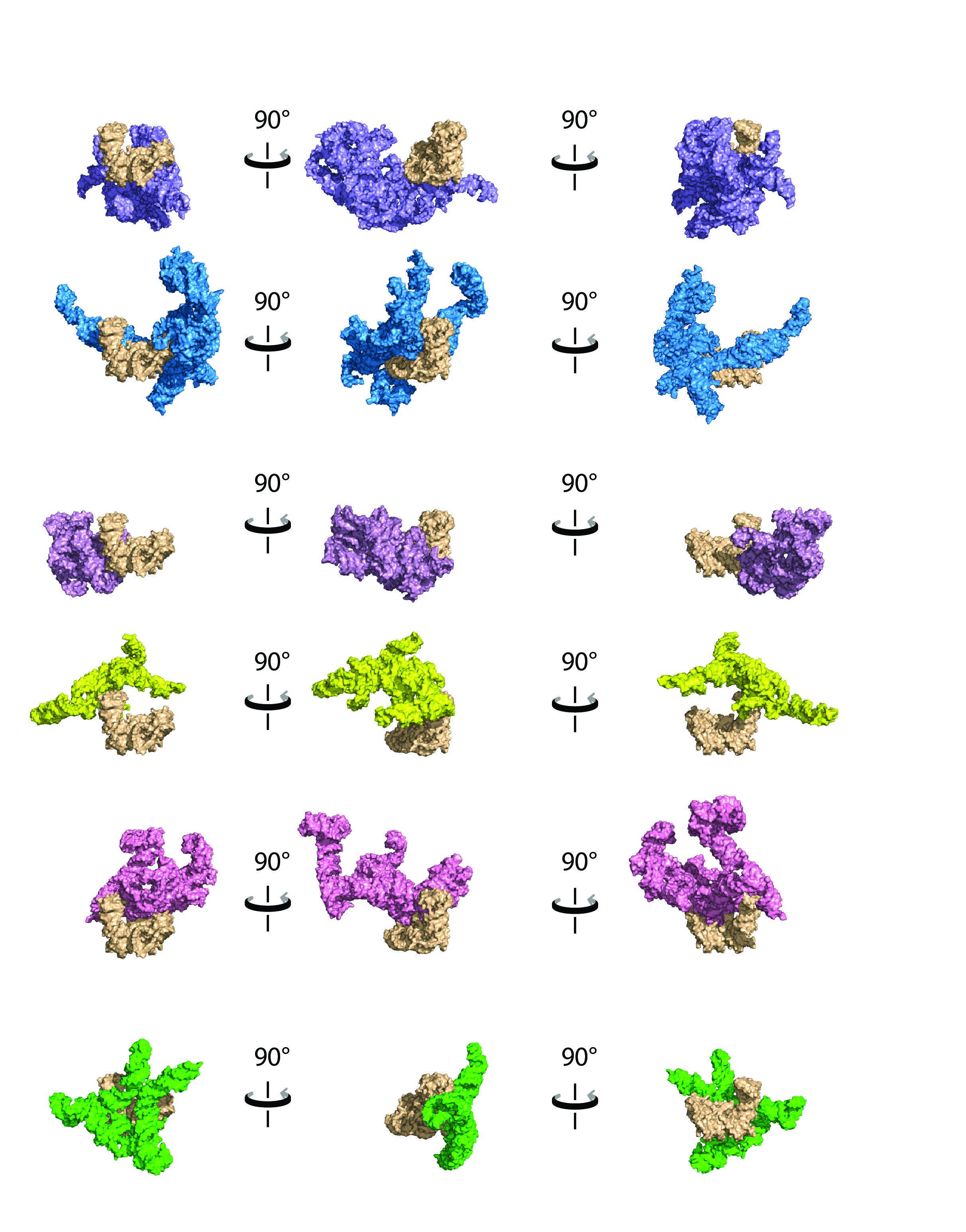


**Figure S2.** Domain 0 of 23S rRNA in association with individual Domains I-VI, rotated around the y-axis. Domains are colored as in Figure 1S.


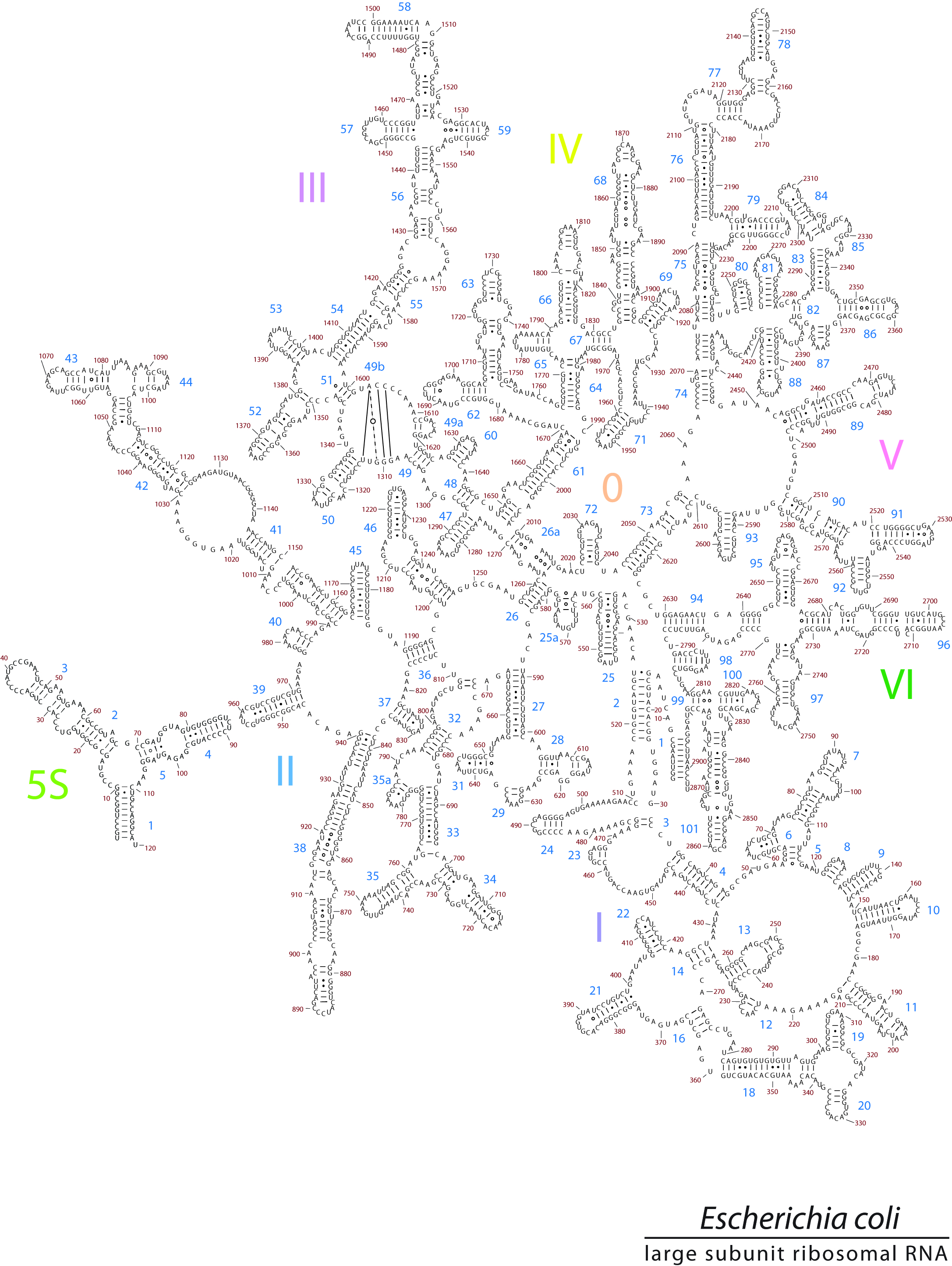


**Figure S3.** 2° Structure^3D^, the revised 2° Structure of the 23S and 5S rRNAs of *E. coli.* The sequence of the 23S rRNA, the helix numbers and the domains are indicated.


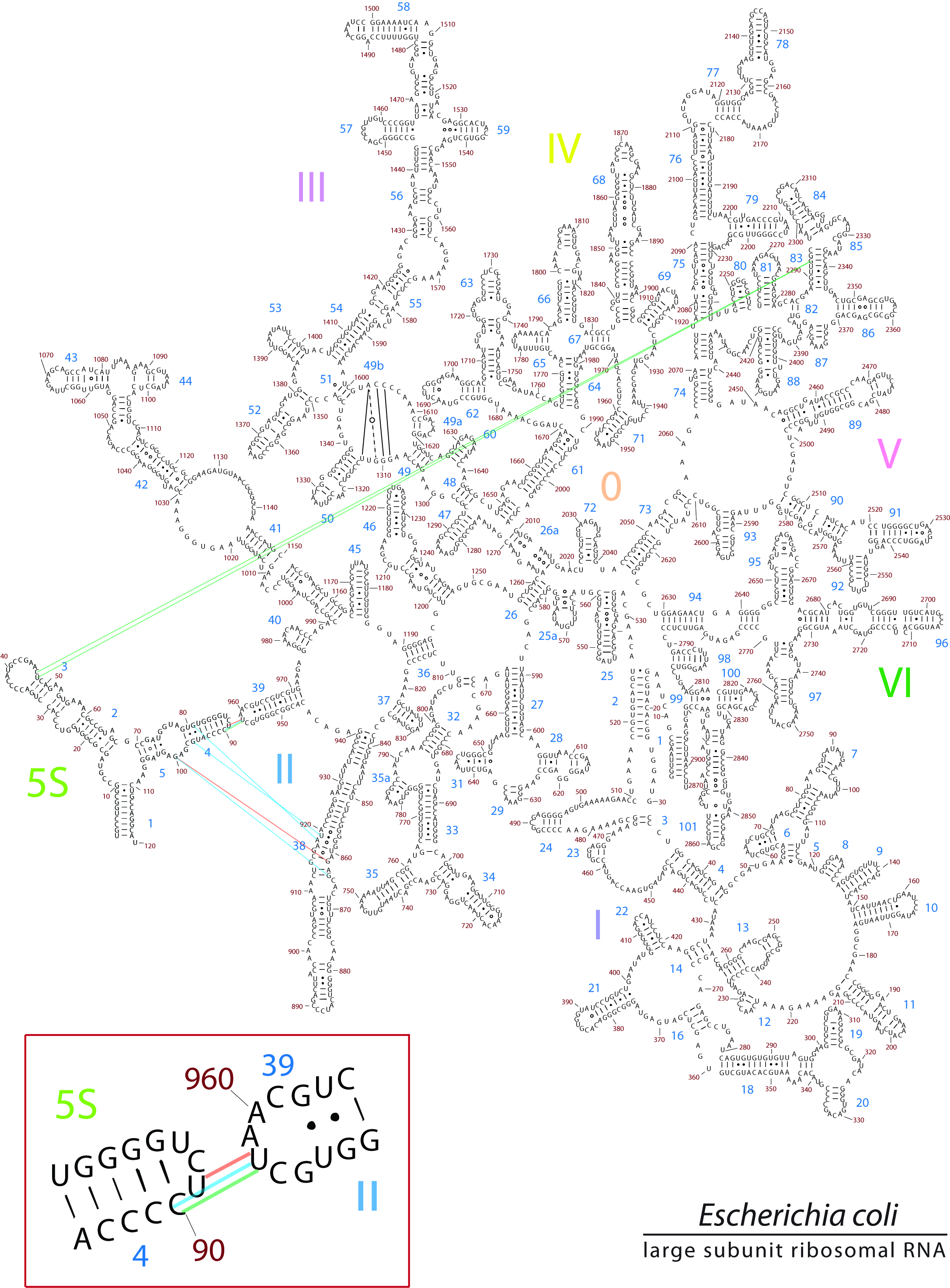


**Figure S4.** 2° Structure^3D^, the revised 2° Structure of the 23S and 5S rRNAs of *E. coli.* The 5S rRNA is located in proximity of Helix 39 of 23S rRNA to reflect their locations in 3D space. Molecular interactions between 23S rRNA and 5S rRNA are shown by lines: stacking (red), phosphate-base (blue), protein mediated (green). The red box depicts an enlarged view of interactions between Helix 39 of 23S rRNA and Helix 4 of 5S rRNA.


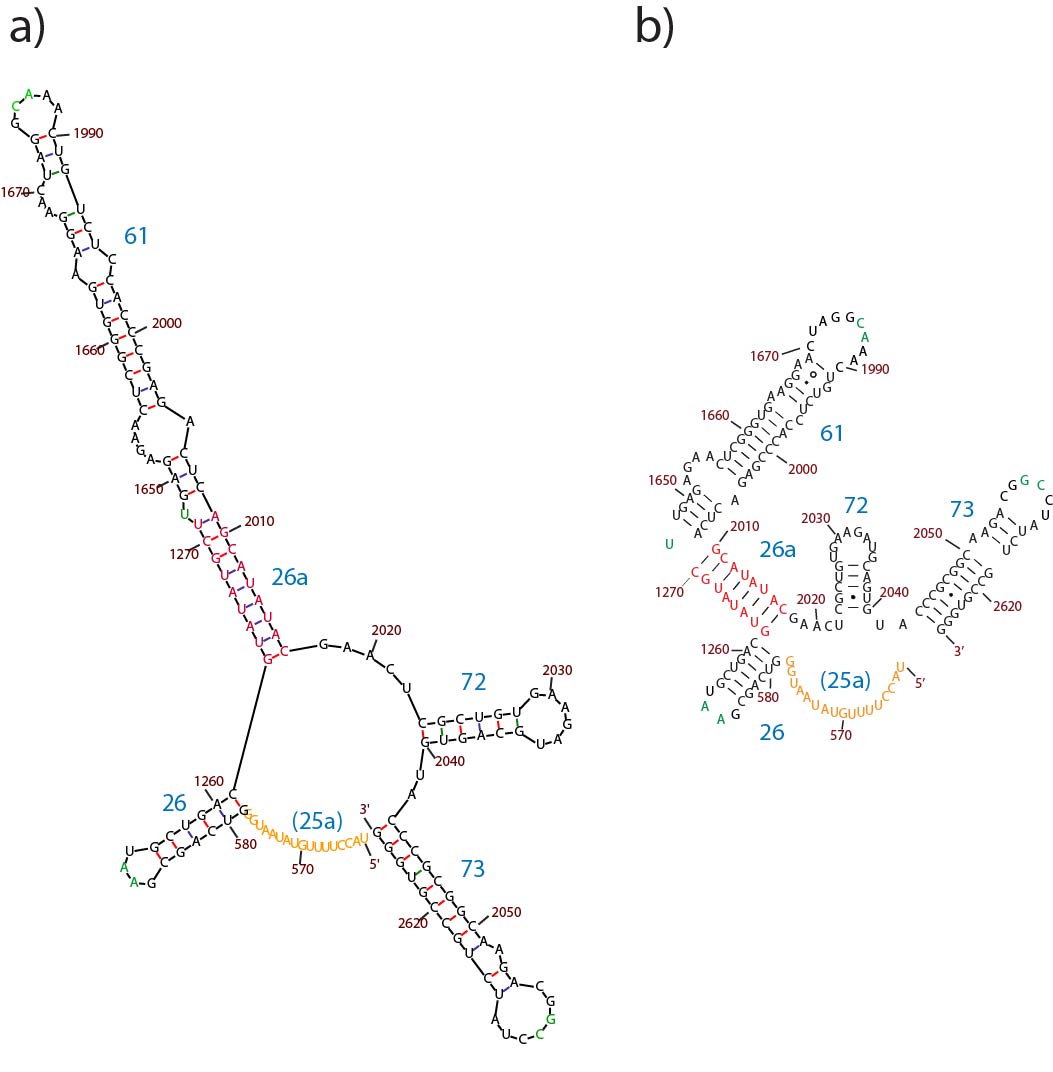


**Figure S5.** 2° Structure of Domain 0 of 23S rRNA predicted by Mfold. a) The representation of folded Domain 0 output by Mfold. b) A representation of folded Domain 0 laid out in a format consistent with 2° Structure^3D^. Linkages of the RNA fragments, added to create a single RNA molecule, are highlighted in green. The modified sequence of Helix 26a is highlighted in red. Helix 25a, unpredicted by Mfold because of non-canonical base pairs, is highlighted in orange.


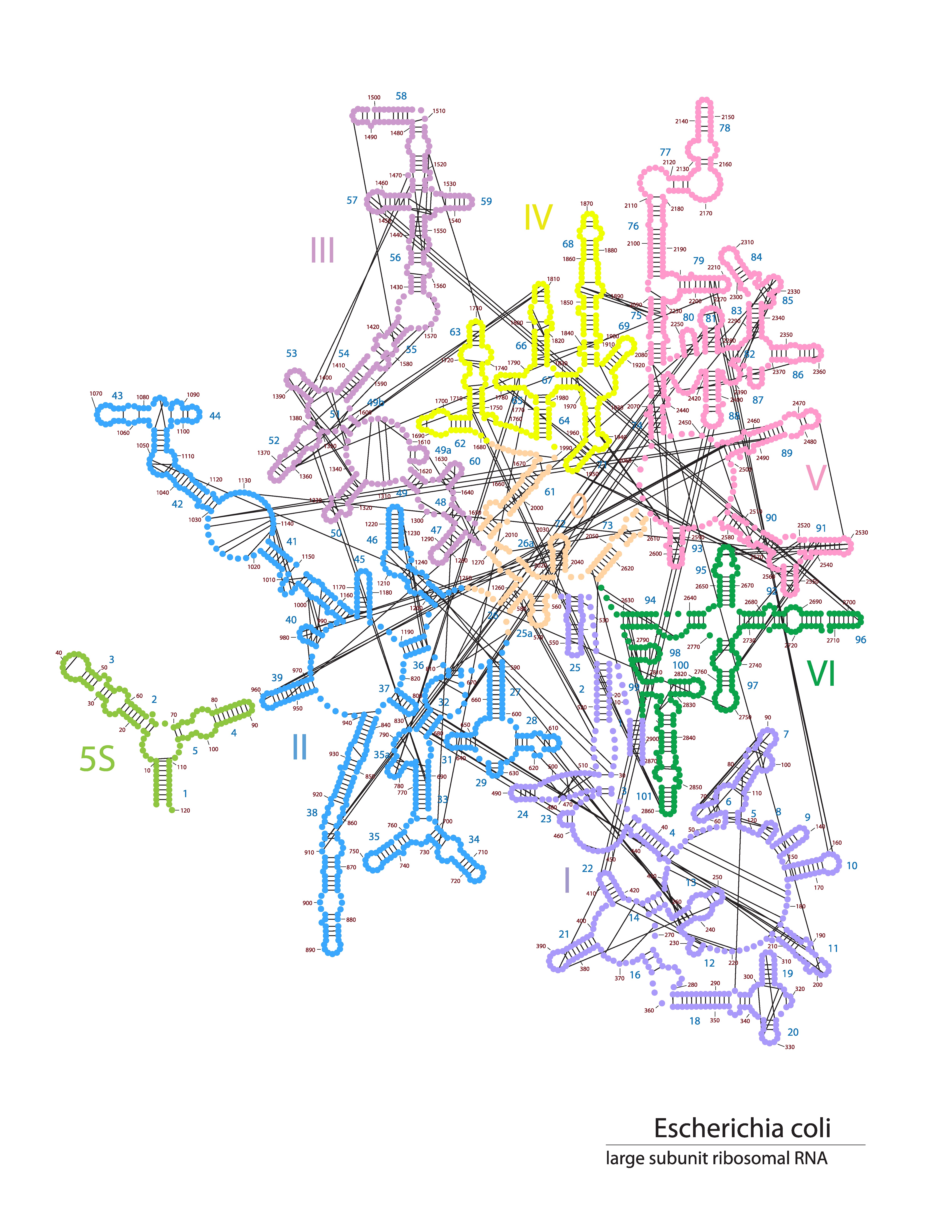


**Figure S6a.** The projection of the four most frequent subtypes of base pair interactions (cWW, tWW, tSS, and cSS) onto 2° Structure^3D^ of 23S and 5S rRNAs. Nucleotides connected by lines in the 2° structure here are base-paired in the 3D structure of the ribosome. The coloring scheme of the domains is the same as in Figure S1.


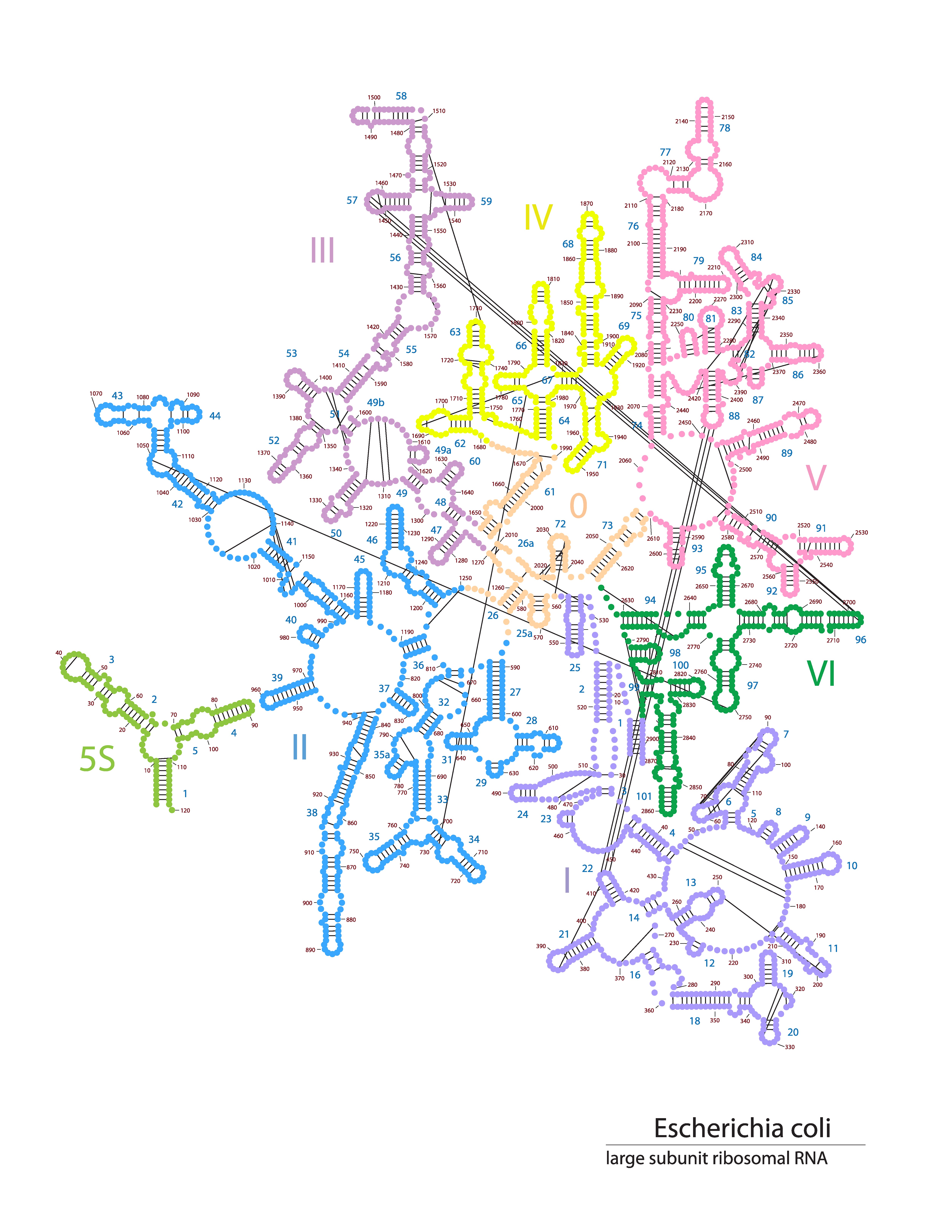


**Figure S6b.** The projection of the cWW base pair interactions onto 2° Structure^3D^ of 23S and 5S rRNAs. Nucleotides connected by lines in the 2° structure here are base-paired in the 3D structure of the ribosome. The coloring scheme of the domains is the same as in Figure S1.


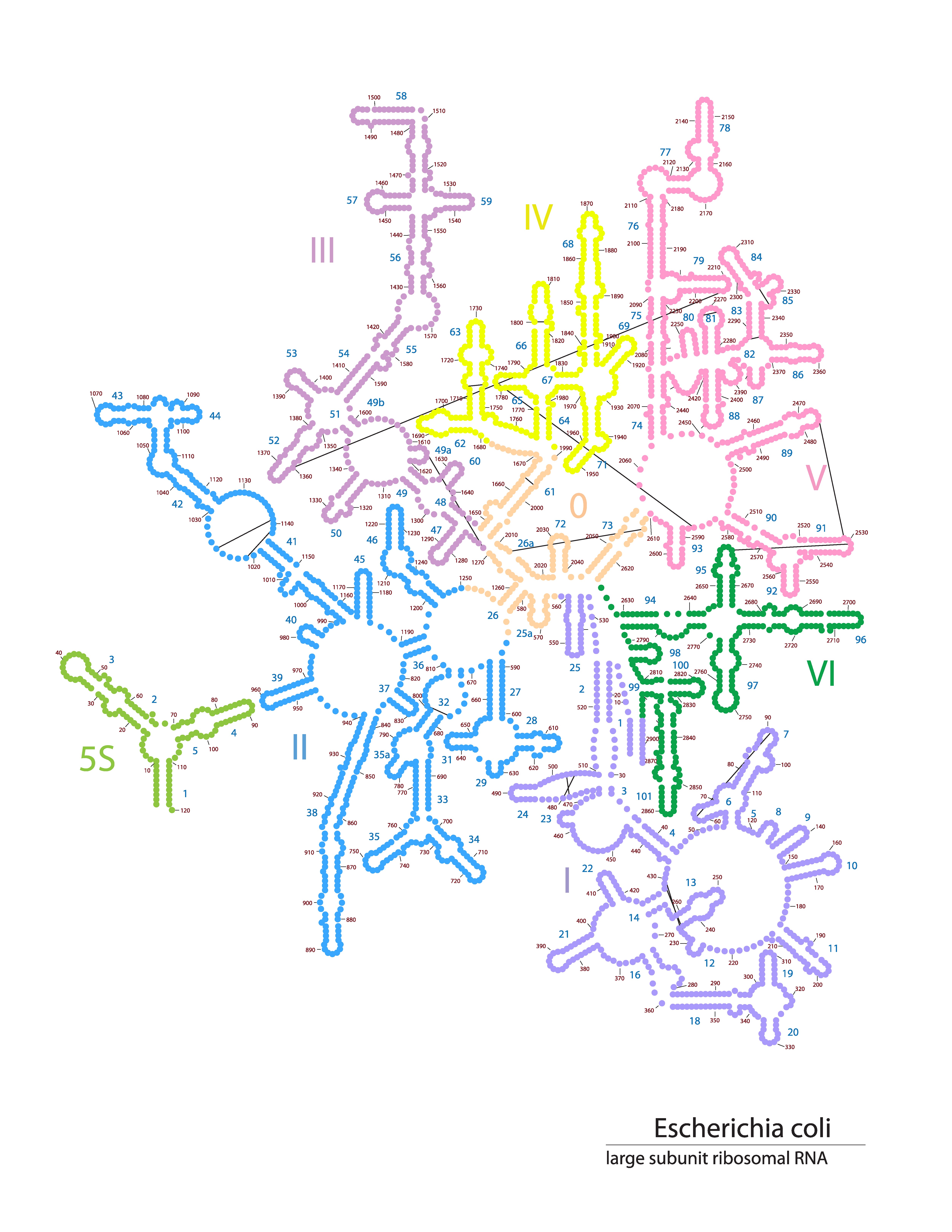


**Figure S6c.** The projection of the tWW base pair interactions onto 2° Structure^3D^ of 23S and 5S rRNAs. Nucleotides connected by lines in the 2° structure here are base-paired in the 3D structure of the ribosome. The coloring scheme of the domains is the same as in Figure S1.


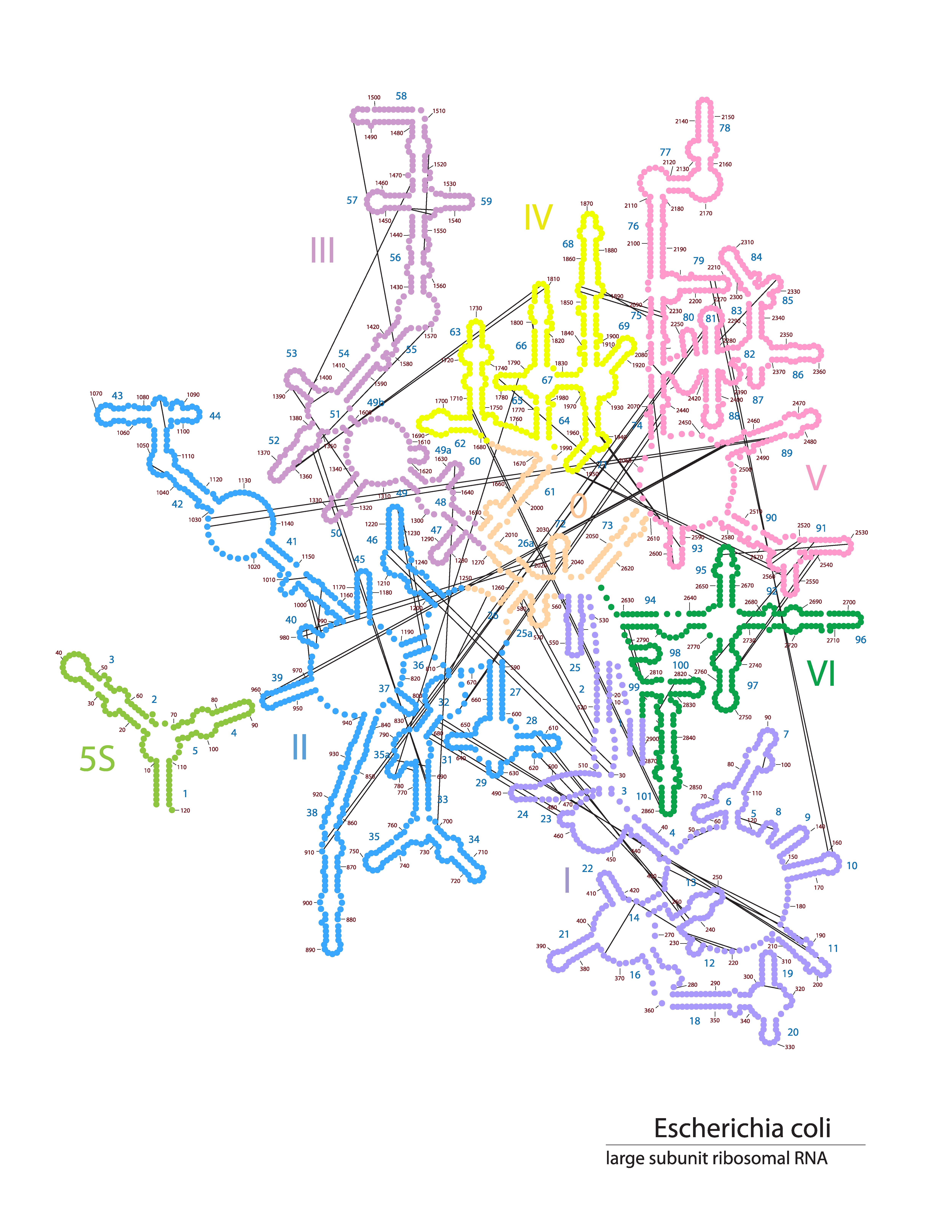


**Figure S6d.** The projection of the cSS base pair interactions onto 2° Structure^3D^ of 23S and 5S rRNAs. Nucleotides connected by lines in the 2° structure here are base-paired in the 3D structure of the ribosome. The coloring scheme of the domains is the same as in Figure S1.


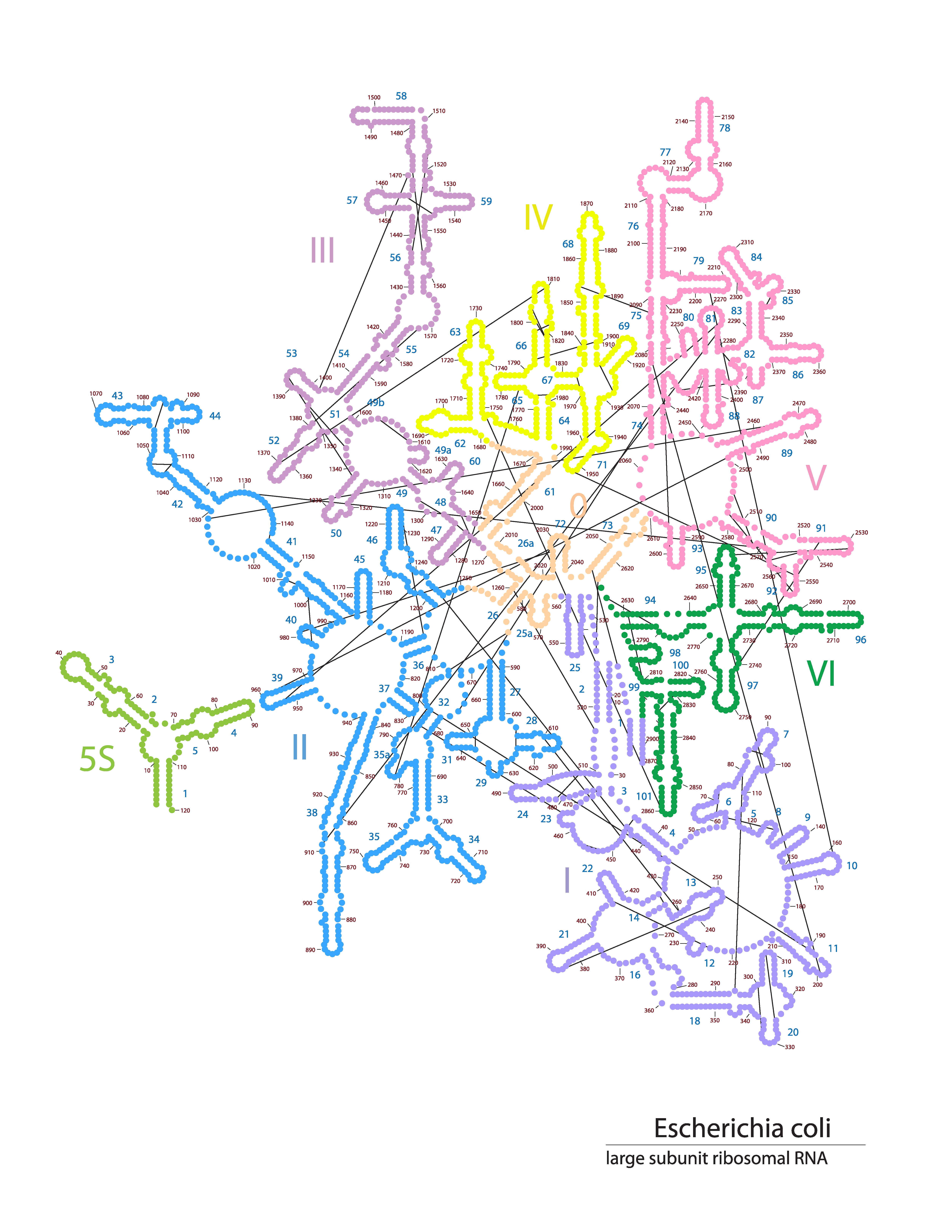


**Figure S6e.** The projection of the tSS base pair interactions onto 2° Structure^3D^ of 23S and 5S rRNAs. Nucleotides connected by lines in the 2° structure here are base-paired in the 3D structure of the ribosome. The coloring scheme of the domains is the same as in Figure S1.


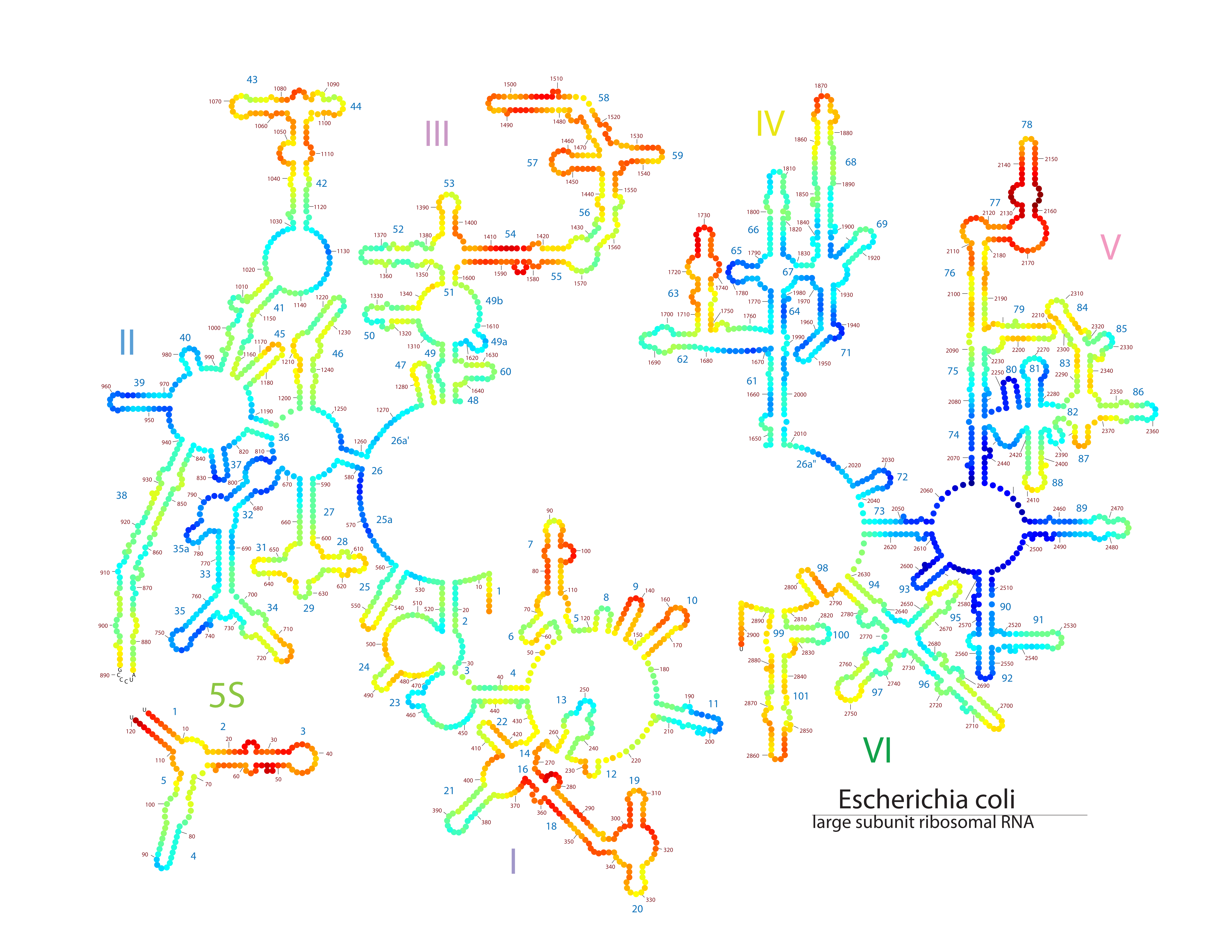


**Figure S7a.** 2° Structure^phylo^ of 23S and 5S rRNAs with nucleotides (represented by circles) colored by their distance from the site of peptidyl transfer. Nucleotides close to the site of peptidyl transfer in the 3D structure are dark blue. Nucleotides that are remote from the PTC in the 3D structure are red.


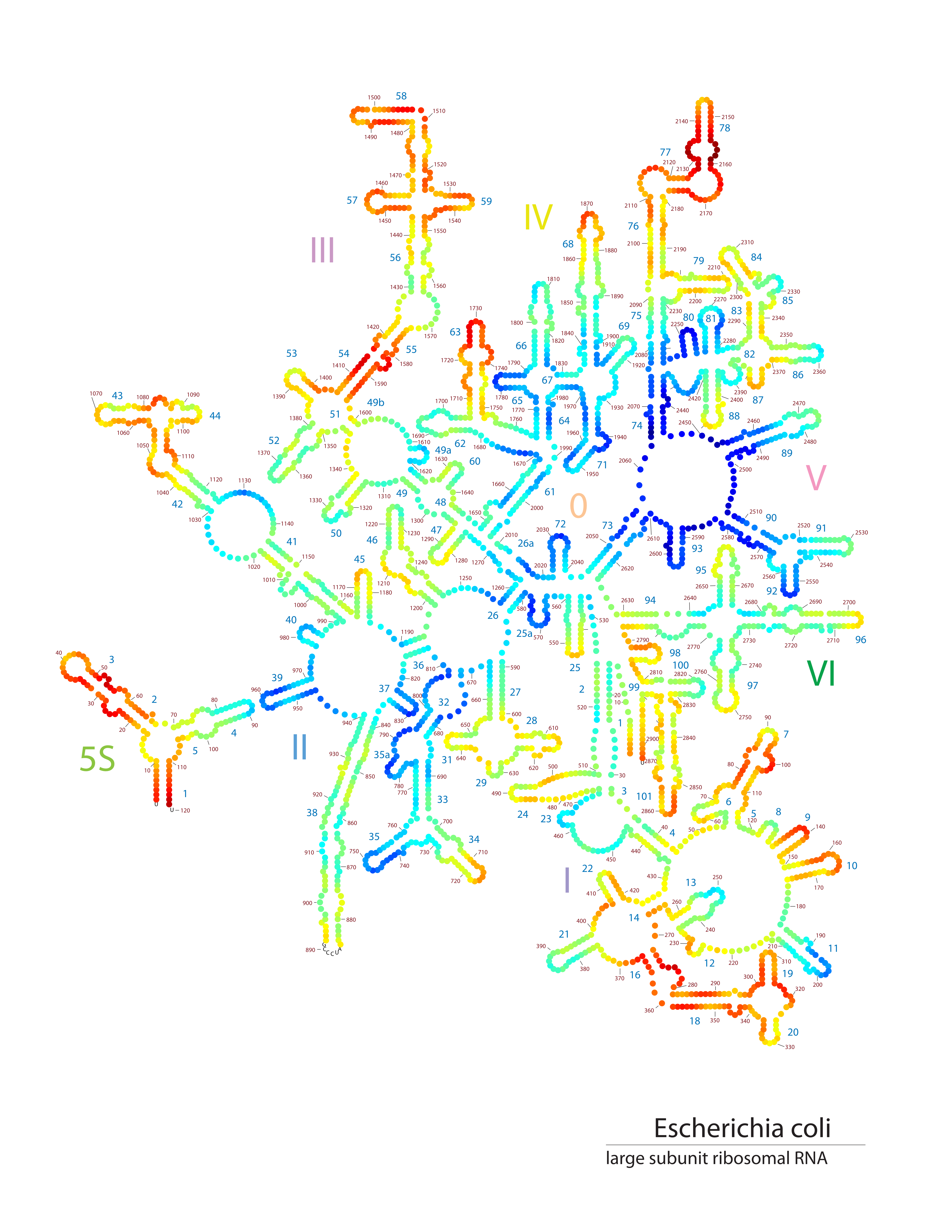


**Figure S7b.** 2° Structure^3D^ of 23S and 5S rRNAs with nucleotides (represented by circles) colored by their distance from the site of peptidyl transfer. Nucleotides close to the site of peptidyl transfer in the 3D structure are dark blue. Nucleotides that are remote from the PTC in the 3D structure are red.
